# Genetic variants of TORC1 signaling pathway affect nitrogen consumption in *Saccharomyces cerevisiae* during alcoholic fermentation

**DOI:** 10.1371/journal.pone.0220515

**Published:** 2019-07-26

**Authors:** Jennifer Molinet, Francisco A. Cubillos, Francisco Salinas, Gianni Liti, Claudio Martínez

**Affiliations:** 1 Departamento de Ciencia y Tecnología de los Alimentos, Universidad de Santiago de Chile (USACH), Santiago, Chile; 2 Millennium Institute for Integrative Biology (iBio), Santiago, Chile; 3 Departamento de Biología, Facultad de Química y Biología, Universidad de Santiago de Chile (USACH), Santiago, Chile; 4 Centro de Estudios en Ciencia y Tecnología de Alimentos (CECTA), Universidad de Santiago de Chile (USACH), Santiago, Chile; 5 Instituto de Bioquímica y Microbiología, Facultad de Ciencias, Universidad Austral de Chile, Valdivia, Chile; 6 Institute for Research on Cancer and Ageing of Nice (IRCAN), Centre National de la Recherche Scientifique (CNRS), INSERM, University of Côte d’Azur, Nice, France; University of Strasbourg, FRANCE

## Abstract

In the alcoholic fermentation process, *Saccharomyces cerevisiae* strains present differences in their nitrogen consumption profiles, these phenotypic outcomes have complex genetic and molecular architectures. In this sense, variations in nitrogen signaling pathways regulated by TORC1 represent one of the main sources of phenotypic diversity in nitrogen consumption. This emphasizes the possible roles that allelic variants from the TORC1 pathway have in the nitrogen consumption differences observed in yeast during the alcoholic fermentation. Here, we studied the allelic diversity in the TORC1 pathway across four yeast strains and determined how these polymorphisms directly impact nitrogen consumption during alcoholic fermentation. Using a reciprocal hemizygosity approach combined with phenotyping under fermentative conditions, we found that allelic variants of *GTR1*, *TOR2*, *SIT4*, *SAP185*, *EAP1*, *NPR1* and *SCH9* underlie differences in the ammonium and amino acids consumption phenotypes. Among these, *GTR1* alleles from the Wine/European and West African genetic backgrounds showed the greatest effects on ammonium and amino acid consumption, respectively. Furthermore, we identified allelic variants of *SAP185*, *TOR2*, *SCH9* and *NPR1* from an oak isolate that increased the amino acid consumption preference over ammonium; representing putative candidates coming from a non-domesticated strain that could be used for genetic improvement programs. In conclusion, our results demonstrated that a large number of allelic variants within the TORC1 pathway significantly impacts on regulatory mechanisms of nitrogen assimilation during alcoholic fermentation.

## Introduction

Wine fermentation is a very complex process whereby yeasts, mainly *Saccharomyces cerevisiae*, convert the sugars present in the must into ethanol, CO_2_ and other metabolites [1,2]. One of the main problems in the wine industry is the low concentration of yeast assimilable nitrogen (YAN) found in grape must [3], where concentrations below 140 mg/L [1] are considered deficient, resulting in an irreversible arrest of the hexose transport. This affects the yeast biomass yield and fermentation efficiency, causing sluggish or stuck fermentations [2,4].

Yeast cells recognize the nature and availability of nitrogen sources and adjust their transcriptional and biosynthetic mechanisms accordingly. When nitrogen is limiting, the cells grow slower, mainly reducing ribosomal biogenesis and translation, resulting in an extension of the cell cycle in G1 [5]. While in the extreme case of nitrogen depletion, cells cease growth and enter in a nitrogen-specific quiescent state [6]. Thus, cells can couple their synthetic capacity and growth rate to the quality and amount of available nitrogen [7]. In this context, nitrogen sources are classified as preferred or non-preferred, depending on their differential assimilation order [8]. This hierarchical preference implies a tight regulation of genes encoding specific enzymes and permeases in response to the nitrogen sources present in the medium [9]. The main mechanisms of nitrogen consumption regulation are: Ssy1-Ptr3-Ssy5 system (SPS), nitrogen catabolic repression (NCR), retrograde signaling pathway (RTG) and the general control of amino acids (GAAC). All these mechanisms are regulated by the TORC1 signaling pathway, representing the main control hub for nitrogen consumption [8,10,11].

TORC1 coordinates cell growth and nutrient availability [12], responding predominantly to the quantity and quality of nitrogen sources present in the medium, likely through the detection of intracellular amino acid levels. The activity of TORC1 decreases following nitrogen starvation and increases upon nitrogen upshift [7]. Two major effector branches function as intermediates between TORC1 activity and several cellular components that affect growth and metabolism: the kinase Sch9 and the Tap42-PP2A complex. In addition to these two proximal effectors, TORC1 modulates distal outputs to positively regulate ribosomal biogenesis and translation, and to inhibit the stress response, which is incompatible with cell growth and is typically induced in quiescent cells [10]. Despite these evidences, there is scarce information on how TORC1 affects the nitrogen consumption profiles in different strains.

During alcoholic fermentation, the preferential nitrogen sources initially internalized correspond to those regulated by the SPS system, followed by nitrogen sources whose transporters are regulated by NCR system [13]. Once the nitrogen sources are assimilated, the majority of them are stored as amino acid pools in the cytoplasm (probably transformed into glutamine) or in the vacuole (positively charged amino acids, such as arginine) [14–16], where once the extracellular nitrogen is depleted, these pools are used for *de novo* synthesis of amino acids or directly incorporated into proteins to initiate cell growth [15,16]. Although all yeasts cells have these mechanisms, *S*. *cerevisiae* strains have a large phenotypic diversity in their nitrogen consumption profiles [17–22]. These differences in nitrogen consumption capacities between strains emerged from the variations in its abilities to uptake specific nitrogen sources. Furthermore, these variations can be due to mutations in the coding sequences of permeases or differential gene expression of nitrogen transporters [17]. Thus, genes encoding for ammonium and amino acid permeases are differentially expressed between strains with differences in nitrogen consumption profiles [16,17,23–26]. Therefore, the uptake of nitrogen sources and their regulation mechanisms become relevant during the alcoholic fermentation. Moreover, QTL (Quantitative Trait Locus) mapping studies in different genetic backgrounds have demonstrated a great diversity of genes participating in the nitrogen consumption phenotype during the alcoholic fermentation [20,22,27,28]. In this sense, Cubillos et al. [20] provided evidence that variations in nitrogen metabolism and signaling, mainly by SPS and NCR systems, were responsible for the differences in nitrogen consumption between *S*. *cerevisiae* strains. However, the molecular mechanisms and the allelic variants of TORC1 pathway modulating nitrogen consumption are unknown. As a result, it is important to understand the role of the allelic diversity in the TORC1 pathway and its relationship to nitrogen consumption during alcoholic fermentation.

The transcriptional analysis of TORC1 activity under fermentative conditions showed numerous genes (more than 300 genes are targets of TORC1), related to nitrogen utilization, which are induced after entry into stationary phase, where nitrogen depletion take place [18,29–31]. Therefore, during alcoholic fermentation yeasts enter to stationary phase as a consequence of nitrogen depletion, inactivating the TORC1 signaling pathway and inhibiting the expression of genes related to biosynthetic pathways and protein synthesis, thereby activating NCR target genes and general stress response [30]. Even though these molecular mechanisms are well understood, how TORC1 influence the expression levels of its target genes and ultimately nitrogen consumption under the alcoholic fermentation context, is a topic of intense research. Thus, we rationalized that differences in nitrogen consumption across *S*. *cerevisiae* isolates could be a result of the allelic diversity present in the TORC1 signaling pathway.

In a previous study [20], we identified a series of QTLs and candidate genes responsible for differences in nitrogen consumption, utilizing a multi-parental yeast population denominated SGRP-4X (Saccharomyces Genome Resequencing Project). Our results suggest that variations in the nitrogen signaling pathways, specifically in the SPS and NCR systems, are responsible for differences in nitrogen consumption between *S*. *cerevisiae* strains. Since the SPS and NCR systems are regulated by TORC1, allelic variants from the TORC1 signaling pathway could be responsible for the differences observed in nitrogen consumption between *S*. *cerevisiae* strains during the alcoholic fermentation. This includes changes in the expression patterns of TORC1 target genes, such as nitrogen permeases. In this study, we took advantage of the SGRP-4X multi-parental population with the aim of identifying genes related to the TORC1 signaling pathway, which affect nitrogen consumption during the alcoholic fermentation and explain the phenotypic variation between *S*. *cerevisiae* isolates. Using a reciprocal hemizygosity approach we identified several alleles affecting the ammonium and amino acids consumption phenotypes. Among the analyzed alleles, the *GTR1* alleles coming from the Wine/European and West African isolates showed the greatest effects on ammonium and amino acid consumption, respectively. Additionally, we identified allelic variants from the YPS128 strain (oak isolate), which positively impacts the nitrogen consumption phenotype by increasing the preference for specific amino acids over ammonium. Overall, our results confirmed that multiple allelic variants related to TORC1 pathway regulate nitrogen assimilation during the alcoholic fermentation.

## Materials and methods

### Strains and culture media

Haploid parental strain DBVPG6765 (WE, Wine/European), DBVPG6044 (WA, West African), YPS128 (NA, North American) and Y12 (SA, Sake) were previously described [32,33]. The complete genotypes are shown in S1 Table. All the strains were maintained on YPDA solid media (2% glucose, 2% peptone, 2% yeast extract, 2% agar).

### Fermentation in synthetic must

Fermentations were carried out in six replicates using synthetic wine must (MS300), mimicking a standard natural must [3,29]. Briefly, MS300 was supplemented with a final concentration of 300 mgN L^-1^ of assimilable nitrogen (YAN) corresponding to 120 mgN L^-1^ of ammonium and 180 mgN L^-1^ of amino acids mixture (612.6 mg L^-1^ L-proline, 503.5 mg L^-1^ L-glutamine, 503.5 mg L^-1^ L-arginine monohydrochloride, 179.3 mg L^-1^ L-tryptophan, 145.3 mg L^-1^ L-alanine, 120.4 mg L^-1^ L-glutamic acid, 78.5 mg L^-1^ L-serine, 75.92 mg L^-1^ L-threonine, 48.4 mg L^-1^ L-leucine, 44.5 mg L^-1^ L-aspartic acid, 44.5 mg L^-1^ L-valine, 37.9 mg L^-1^ L-phenylalanine, 32–7 mg L^-1^ L-isoleucine, 50.0 mg L^-1^ L-histidine monohydrochloride monohydrate, 31.4 mg L^-1^ L-methionine, 18.3 mg L^-1^ L-tyrosine, 18.3 mg L^-1^ L-glycine, 17.0 mg L^-1^ L-lysine monohydrocloride, and 13.1 mg L^-1^ L-cysteine). The strains were initially grown under constant agitation in 5 mL of MS300 during 24 hours at 25°C. Next, 1 x 10^6^ cells mL^-1^ were inoculated into 12 mL of MS300 (using 15 mL conical tubes) and incubated at 25°C, with no agitations for 20 days. CO_2_ production was monitored by daily weighing the tubes and determining weight loss over the time course of the fermentation. The CO_2_ loss curves were fitted to a sigmoid non-linear regression [34] and the kinetic parameters determined were: the maximal CO_2_ production rate (V_max_), V_50_/V_max_ ratio and efficiency, previously described by Marullo et al. [35].

### Determination of nitrogen consumption

On the sixth day of fermentation, stage at which most nitrogen consumption differences can be observed [22,34], 12 mL of synthetic wine must (MS300) were centrifuged at 9000x*g* for 10 min and the supernatant was collected. The concentration of ammonium and the 19 amino acids present in the must were determined by derivatization with DEEMM [36] and separation by HPLC using a Bio-Rad HPX-87H column in a Shimadzu Prominence HPLC equipment (Shimadzu, USA) [37]. The consumption of each nitrogen source was defined as the difference between the initial concentration and the concentration determined at day six of fermentation.

### Selection of candidate genes

The candidate genes were selected from a previous QTL mapping study [20] and obtained using a four-parental yeast population (SGRP-4X). Genomic regions comprising 30 kb around of the selected QTL were examined in the *Saccharomyces Genome Database* (https://www.yeastgenome.org/) for candidate genes, selecting genes with a direct function in the TORC1 pathway (Table 1). The sequences of the candidate genes were downloaded from the SGRP (Saccharomyces Genome Resequencing Project) BLAST server using the information described by Bergström et al. [38] (http://www.moseslab.csb.utoronto.ca/sgrp/), aligned using MUSCLE (http://www.ebi.ac.uk/Tools/msa/muscle/) and its polymorphisms were analyzed by SIFT (Sorting Intolerant from Tolerant) [38,39].

**Table 1:** Candidate genes selected for validation by reciprocal hemizygosity analysis.

QTL	Phenotype	Position QTL	p-value (-log10)	Gene	Description^a^
9	Lysine	X: 235972	8.717	*SAP185*/YJL098W	Protein that forms a complex with the Sit4 protein phosphatase; required for Sit4 function
12	Methionine/Aspartate	XI: 54205	12.065	*EAP1*/YKL204W	eIF4E-associated protein, competes with eIF4G for binding to eIF4E; plays a role in cell growth, implicated in the TOR signaling cascade
12	Methionine/Aspartate	XI: 54205	12.065	*TOR2*/YKL203C	PIK-related protein kinase and rapamycin target; subunit of TORC1 and TORC2
20	Tryptophane	XIV: 281995	10.304	*NPR1*/YNL183C	Protein kinase; stabilizes several plasma membrane amino acid transporters; its activity is negatively regulated via phosphorylation by TORC1
NA	Glutamate	IV:371305	9.533	*SIT4*/YDL047W	Ceramide-activated, type 2A-related serine-threonine phosphatase; functions in G1/S transition of mitotic cycle
NA	Arginine	VIII: 505776	11.188	*SCH9*/YHR205W	AGC family protein kinase; phosphorylated by Tor1 and required for TORC1-mediated regulation of ribosome biogenesis, translation initiation, and entry into G0 phase
NA	Ammonium	XIII: 22769	9.791	*GTR1*/YML121W	Subunit of a TORC1-stimulating GTPase complex (EGOC); subunit of the heterodimeric Gtr1-Gtr2 GTPase complex that stimulates TORC1 in response to amino acid stimulation

^a^ Descriptions obtain from *Saccharomyces* Genome Database (SGD).

NA: Non-Assigned. QTL not considered by Cubillos et al. [20].

### Reciprocal hemyzigosity assay

A reciprocal hemizygosity assay was performed to validate the candidate genes [40]. Briefly, we used haploid parental strains for the deletion of each target gene using *URA3* as selectable marker, constructing all possible combinations of single deletions. Then, the parental strains carrying the deletions were crossed to generate the reciprocal hemizygote strains, which were selected in double drug plates (300 μg mL^-1^ Hygromycin B and 100 μg mL^-1^ Nourseothricin). The deletions of the target genes were confirmed by PCR using the primer pairs A1/S8 or A4/S5 (S2 Table) [41]. The parental strains utilized in the reciprocal hemizygosity assay were chosen using a criterion: initially, we grouped the segregants from the SGRP-4X population using the genotype information of the mapped region (QTL); then, we used the phenotypic information (nitrogen consumption) to sort the segregants; and afterward, we chose for the reciprocal hemizygosity assay the parental genotype showing the higher phenotypic differences respect to the other three parental strains. Finally, the selected parental strain was crossed against the other three genetic backgrounds, and these reciprocal hemizygote strains were fermented in synthetic must and its nitrogen consumption profiles were determined.

### Determination of growth variables

Micro-cultivation experiments were performed in synthetic wine must MS300 at 25°C for 48–72 hours until all strains reached stationary phase. The strains were pre-grown in MS300 medium at 28°C for 24 hours. Pre-cultures were diluted at an initial OD_600nm_ of 0.1 and used to inoculate a 96 well plate with a final volume of 200 μL. The OD_600nm_ was monitored every 20 minutes in a Tecan Sunrise absorbance microplate reader (Tecan Group Ltd., Switzerland). All the experiments were performed in triplicate. Lag phase, doubling time, growth efficiency and the maximum specific growth rate (μ_max_) were estimated as previously described [42,43]. For this, the parameters were determined following curve fitting (OD values were transformed to ln) with the Gompertz function [44] using the R software version 3.3.2. The doubling time was obtained as log (2) divided by μ_max_.

### RNA extraction and qPCR assay

RNA extractions were performed in triplicate from cultures grown in synthetic must (MS300). Briefly, samples were collected by centrifugation at 6, 24 and 48 hours of fermentation. Cultures were harvested by centrifugation and cells were treated with 10 units of Zymolyase (Seikagaku Corporation, Japan) for 30 min at 37°C. RNA was extracted utilizing the E.Z.N.A Total RNA Kit I (OMEGA) according to the instructions. RNA samples were then treated with DNase I (Promega, USA) to remove genomic DNA traces and total RNA was recovered using the GeneJET RNA Cleanup and Concentration Micro Kit (Thermo Scientific). Purified RNA concentrations were determined using an UV-Vis spectrophotometer EPOCH equipment (BioTeK Instruments Inc., USA) and verified by 1.5% agarose gels.

The cDNA was synthesized using one unit of M-MLV Reverse transcriptase (Promega), 0.4 μg of Oligo (dT)_15_ primer and 0.8 μg of RNA in a final volume of 25 μL according to the instructions. The cDNA samples obtained were quantified using an UV-Vis spectrophotometer EPOCH equipment (BioTeK Instruments Inc., USA). The qPCR reactions were carried out using HOT FIREPol EvaGreen qPCR Mix Plus (Solis BioDyne) in a final volume of 20 μL, containing 0.25 μM of each primer and 1 μL of the cDNA previously synthesized. The qPCR reactions were carried out in two replicates using a Step One Plus Real-Time PCR System (Applied Biosystems, USA) under the following conditions: 95°C for 15 min and 40 cycles at 95°C for 15 s, 55°C for 15 s and 72°C for 15 s. The genes and primers used are listed in S2 Table. The relative expression of each gene was quantified using the mathematical method described by Pfaffl [45] and normalized with three housekeeping genes according to Vandesompele et al. [46]. The housekeeping genes *ACT1*, *UBC6* and *RPN2* were previously described by Teste et al. [47]. The ΔCt were analyzed using the Mood test, a nonparametric test was used to analyze the equality of medians when the sample sizes are small [48].

### Gene Ontology (GO) analysis

GO analysis was performed using the tools provided by WebGestalt (WEB-based Gene SeT AnaLysis ToolKet), which groups genes into different categories according to Biological Process using the GO Slimm Mapper and also determines statistically enriched GO terms [49].

### Statistical analysis

The enological parameters were compared using an analysis of variance (ANOVA) and the mean values of the experiments were statistically analyzed using Tukey’s multiple comparisons test and Student’s t-test. In case of multiple comparisons, data was analyzed using Student’s t-test and corrected using Bonferroni method. Probability values lower than 0.05 (P<0.05) were considered as statistically significant.

## Results

### Selection of candidate genes connecting nitrogen consumption with the TORC1 pathway

To determine the role of the TORC1 signaling pathway in the nitrogen consumption profiles across *S*. *cerevisiae* strains, 26 QTLs obtained from a previous study [20] for 15 different nitrogen sources (14 amino acids and ammonium) were analyzed. Previously, allelic variants in four genes related with nitrogen metabolism (*CPS1*, *ASI2*, *LYP1* and *ALP1*) were identified as responsible for differences in arginine consumption and variations in the SPS and NCR pathways [20]. Considering that the SPS and NCR systems are under TORC1 control, we looked for genes participating in the TORC1 pathway within the same set of 26 QTLs.

**Fig 1:**
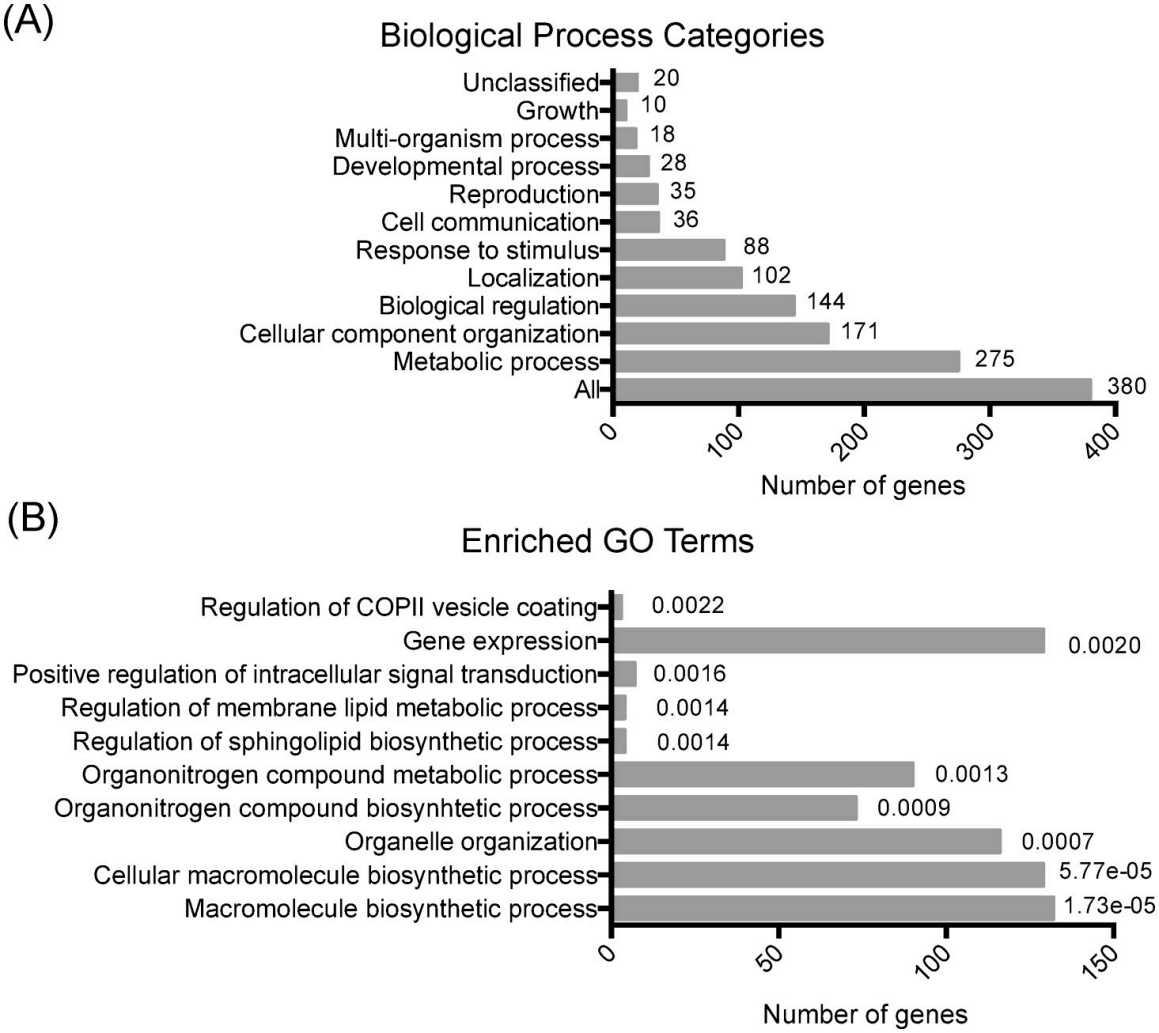
GO term enrichment for genes present in the QTLs genomic intervals. The GO classification (A) and enrichment analysis (B) for the 380 genes present in 26 QTLs identified by Cubillos et al. [20]. In panel A, the values represent the number of genes for each category. In panel B, the P values for each enrichment category are shown (Bejamini-Hochberg procedure).

Initially, we investigated functional gene enrichment categories by using the Gene Ontology (GO) database [49]. This analysis revealed that 380 genes, within the 26 genomic intervals of the QTLs, were mainly divided into 10 different categories according to Biological Process using the GO Slimm Mapper (Fig 1A), which maps the annotations for a group of genes in general terms. Most of these genes (275 genes) were grouped in the “Metabolic Process” category, while 144, 88 and 36 genes were grouped in the “Biological Regulation”, “Response to Stimulus” and “Cell Communication” categories, respectively (Fig 1A). We also determined categories with significantly enriched in GO process (Fig 1B); for instance, “positive regulation of intracellular signal transduction” (p = 0.0016), “organonitrogen compound biosynthetic process” (p = 0.0009) and “organonitrogen compound metabolic process” (p = 0.0013). Genes grouped in these categories participate mainly in the TORC1, MAPK and PKA signaling pathways, confirming that the mapped QTLs contain genetic variants participating in nitrogen signaling pathways and nitrogen metabolism.

From the group of genes related to the nitrogen metabolism and regulation, four genes were previously validated (*CPS1*, *ASI2*, *ALP1* and *LYP1*) [20], and seven new candidate genes were directly related to the TORC1 signaling pathway (Table 1). These genes were selected from QTLs identified for lysine, aspartic acid, arginine, tryptophan, glutamic acid and ammonium. The candidate genes selected were: *SAP185*, which encodes a protein required for Sit4p function in the phosphatase complex, located 7 Kb near the QTL9 peak (lysine consumption phenotype) in chromosome X; *EAP1* and *TOR2*, genes encoding an eIF4E-associated protein and PIK-related protein kinase, respectively, both located in the QTL12 peak (aspartic acid consumption phenotype) in chromosome XI; and *NPR1* gene encoding a protein kinase, located 11 Kb near the QTL20 peak (tryptophan consumption phenotype) in chromosome XIV. Moreover, we considered three more QTLs not previously considered by Cubillos et al. [20] identified for glutamic acid, arginine and ammonium consumption (Table 1). The glutamic acid peak in chromosome IV is 600 bp near *SIT4*, which encodes a type 2A-related serine-threonine phosphatase; the arginine peak in chromosome VIII is 3.6 Kb near *SCH9*, an AGC family protein kinase; and the ammonium peak in chromosome XIII is 4 Kb near *GTR1*, gene encoding a subunit of a TORC1-stimulating GTPase complex (S1 Fig). Furthermore, all these genes present SNPs in the coding and regulatory region (S3 Table).

**Fig 2:**
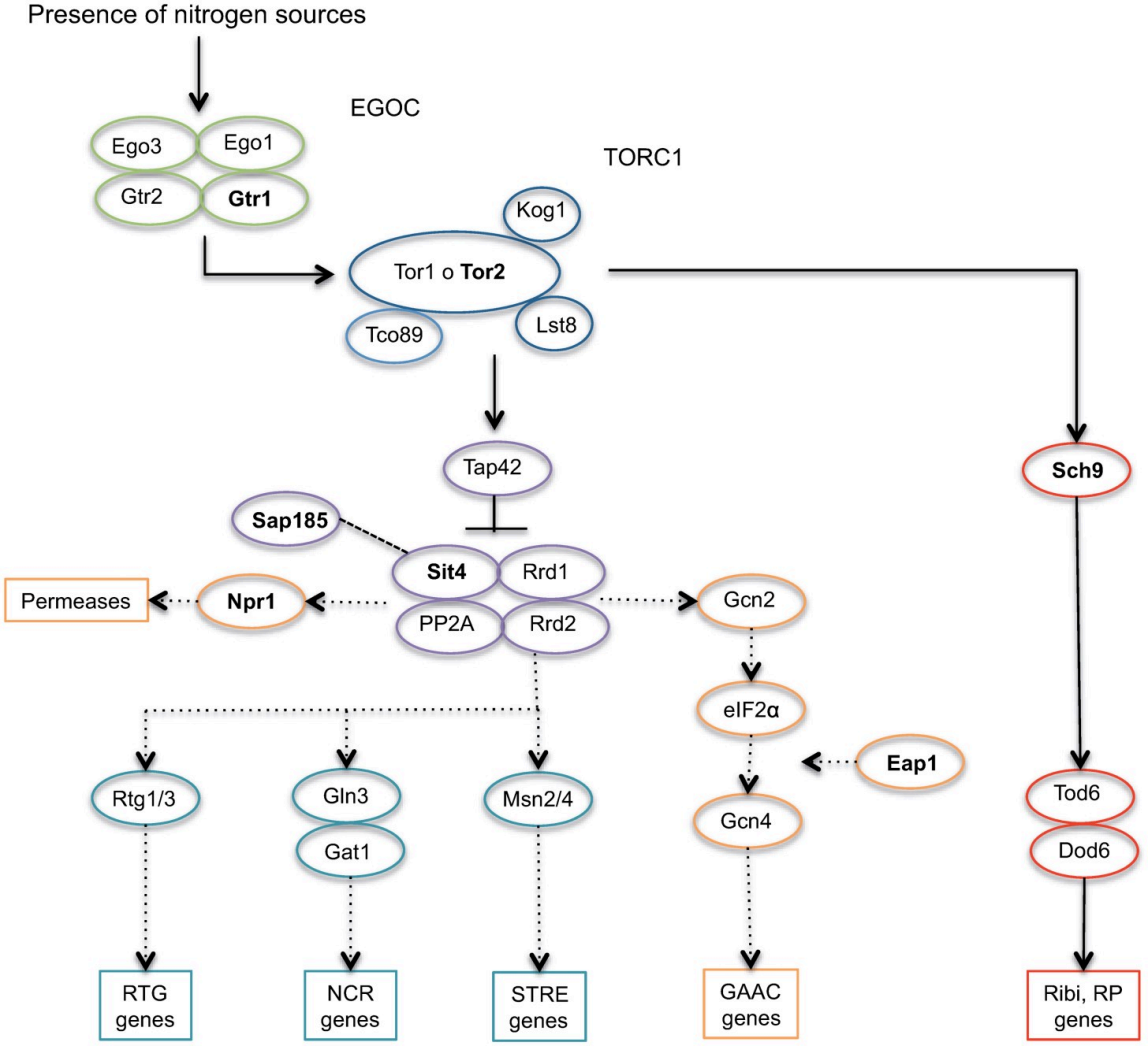
Selection of candidate genes associated to TORC1 pathway. In presence of nitrogen sources the EGO complex is activated, stimulating the activation of TORC1, which phosphorylates its two proximal targets: Sch9 and phosphatase complex. The phosphorylation of Sch9 implies the expression of genes related to ribosomal proteins and ribosomal biogenesis. The phosphorylation of the phosphatase complex promotes its inactivation and the repression of genes related with nitrogen metabolism (RTG, NCR and GAAC) and stress response. The genes selected in this study are in bold.

In summary, we selected seven genes participating at different stages of the TORC1 signaling pathway (Fig 2): *GTR1* from EGOC, which regulates the TORC1 activity; *TOR2*, kinase of TORC1; *SCH9*, proximal TORC1 effector; *SIT4* and *SAP185* from the phosphatase complex, proximal TORC1 effectors; and, *NPR1* and *EAP1*, distal outputs downstream of TORC1.

### Allelic variants underlying differences between strains for nitrogen consumption

In order to determine the effect of each allelic variant on nitrogen consumption during alcoholic fermentation, we performed a reciprocal hemizygosity analysis. For each gene, we selected the parental strain for which the segregants from the SGRP-4X population showed the most important difference in nitrogen consumption for the QTL region mapped (S2 Fig). Then, we crossed this parental strain against the other three strains. Hence, we evaluated each gene in three different crosses. The parental WA was chosen for the genes *SAP185*, *SCH9* and *GTR1*, parental SA for the genes *TOR2* and *EAP1*, parental WE for the *NPR1* gene, and parental NA for the *SIT4* gene (S2 Fig).

**Fig 3:**
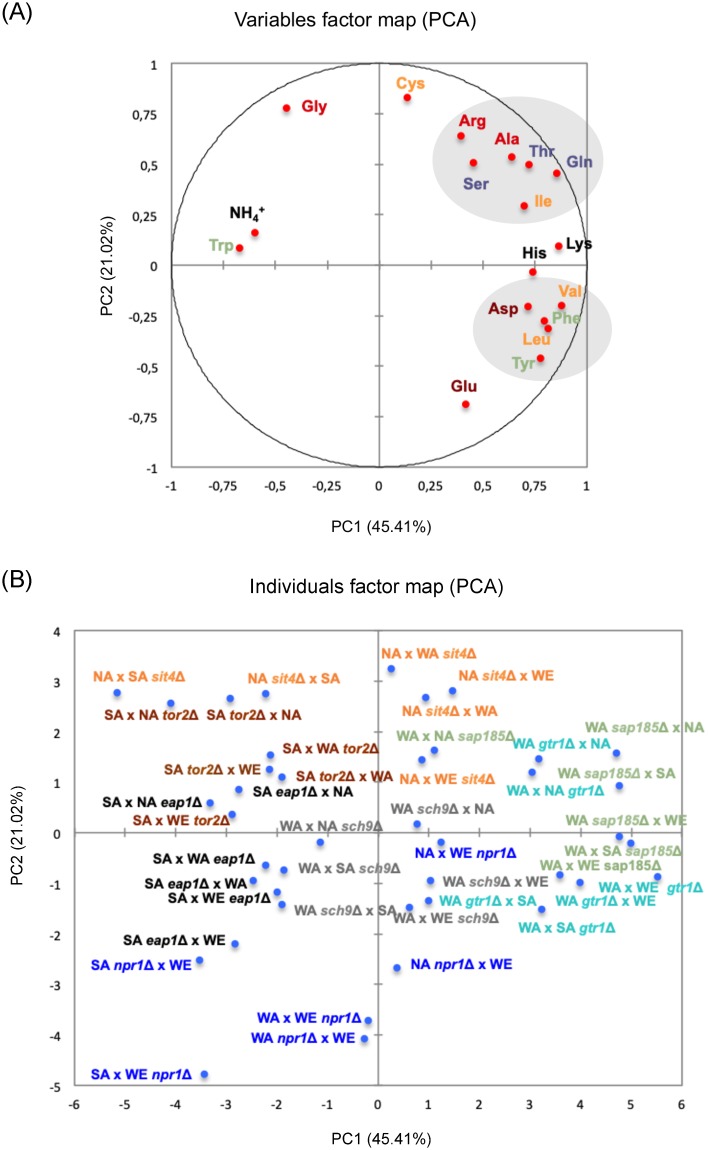
Principal component analysis (PCA) for nitrogen sources consumption and the hemizygous strains. PCA for ammonium and amino acids consumption (A) and individual reciprocal hemizygotes (B). In panel (A), colors of the amino acids represent their respective transporters: Dip5 in brown; Agp1 and Gnp1 in blue; Gap1 in red; Tat1 and Tat2 in green; Bap2 and Bap3 in orange; Hip1 and Lyp1 in black. In panel (B), colors of the strains represent each candidate gene analyzed. Light Blue: *GTR1*; green: *SAP185*, gray: *SCH9*; brown: *TOR2*; orange: *SIT4*; blue: *NPR1*; and black: *EAP1*. The shadow regions in panel (A) represent groups of amino acids for which its consumption co-correlate.

The hemizygous strains showed statistically significant differences (p < 0.05, using Student’s t-test and Bonferroni correction) in the consumption of at least one nitrogen source for the seven genes analyzed, determined as the YAN difference observed between the beginning and day six of fermentation in synthetic must (S4–S11 Tables). In order to address how the phenotypic differences between hemizygous strains correlate with the consumption of nitrogen sources, we performed a global Principal Component Analysis (PCA) using this information (Fig 3). The PC1 and PC2 components explain 45% and 21% of the observed variation, respectively; and combined, the two variables explain 66% of the overall variation. The PCA for nitrogen consumption shows two groups of amino acids (Fig 3A). The first group was made up of arginine, serine, alanine threonine, glutamine and isoleucine, which correspond to amino acids with polar uncharged, non-polar and polar positively charged side chains. The second group contained valine, aspartic acid, phenylalanine, leucine and tyrosine, which correspond to amino acids with polar negatively, aromatic and non-polar side chain. Furthermore, we observed as common feature of each group, the nitrogen transporters utilized by these amino acids, where the first group corresponded to amino acids transported by Gap1p, Agp1p and Gnp1p, while in the second group of amino acids were transported by Bap2p, Bap3p, Tat1p and Tat2p. Importantly, the PCA also indicated that ammonium consumption correlates negatively with amino acid consumption (Fig 3A). Similarly, the amino acids within the same group co-correlate, showing a significant Spearman correlation, and suggesting a concerted consumption of amino acids from the same group (p < 0.05, S3 and S4 Figs). Finally, the individuals factor map allowed the separation of the hemizygous strains according to the gene analyzed but not by genetic backgrounds (Fig 3B), grouping strains with *GTR1*, *SAP185* and *SIT4* allelic variants (except for NA x SA cross), and strains with *TOR2*, *EAP1* and *NPR1* allelic variants (except for WA x WE cross). Altogether, the results confirmed the effect of the analyzed genetic variants in the nitrogen consumption phenotype under fermentation conditions.

### Allelic variants related to TORC1 pathway preferentially impact ammonium consumption

**Fig 4:**
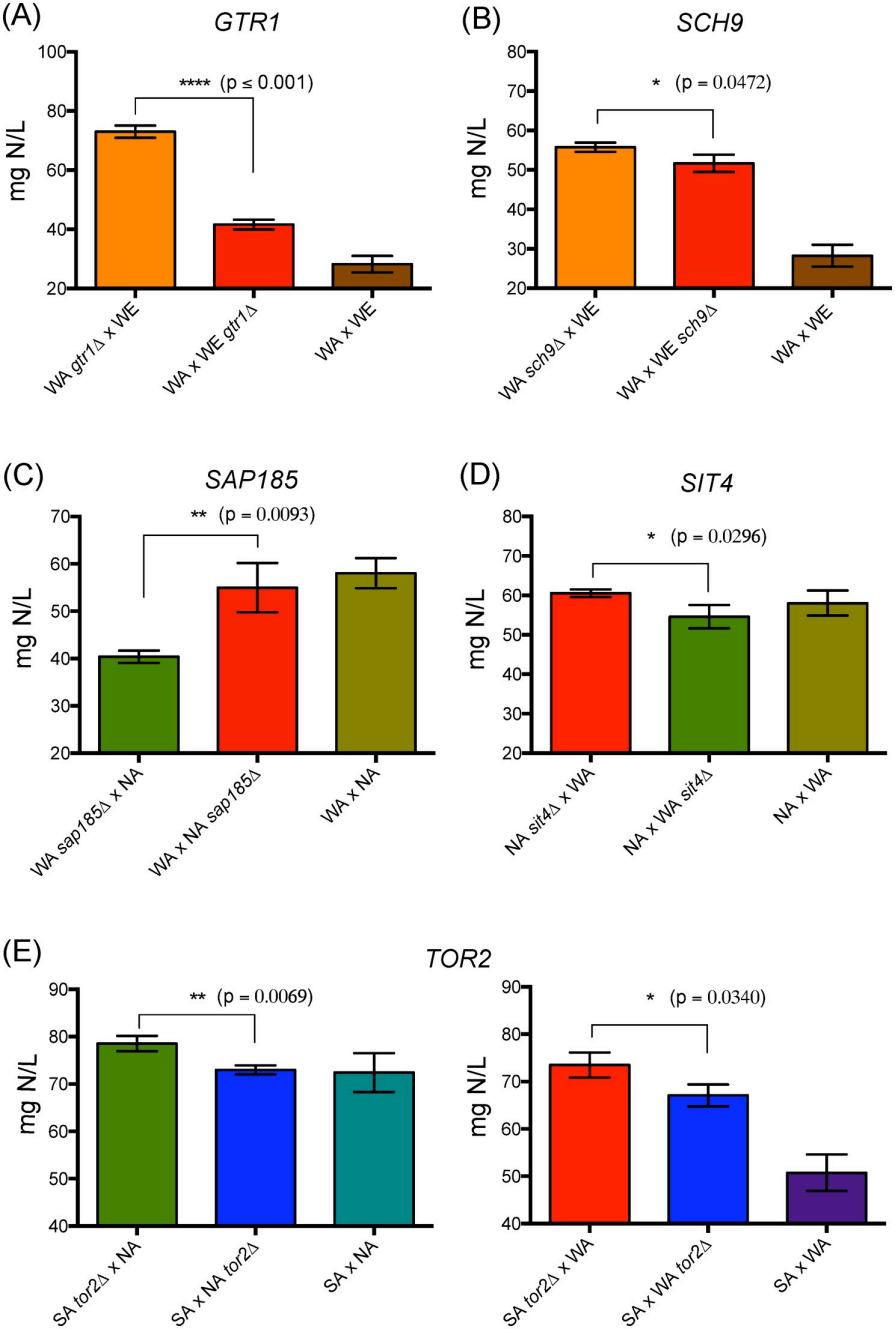
Reciprocal hemizygosity analysis for alleles affecting the ammonium consumption phenotype. Ammonium consumption for *GTR1* (A) and *SCH9* (B) reciprocal hemizygous strains, cross WA x WE; *SAP185* (C) and *SIT4* (D) reciprocal hemizygous strains, cross NA x WA; *TOR2* reciprocal hemizygous strains, cross SA x NA and SA x WA (E). Plotted values correspond to the average of three biological replicates, with their standard deviation represented by bars (mean ± SD). The asterisks represent different levels of significance between the phenotypes of the hemizygous strains (t-test; * p<0.05, ** p<0.01, *** p<0.001 and **** p<0.0001).

Ammonium represents the main nitrogen source in wine must [1]. Across the genes evaluated, we observed significant differences for ammonium consumption in the hemizygous strains for *GTR1*, *SCH9*, *TOR2*, *SAP185* and *SIT4* (Fig 4). The hemizygous strains carrying the WE allele for *GTR1* and *SCH9* had a higher ammonium consumption (43% and 7.3%, respectively) when compared to the WA allele (Fig 4A and 4B). *GTR1* showed the greatest effect in ammonium consumption and significantly impacted the overall YAN, with a difference in consumption of 15% between hemizygous strains (S4 Table). Conversely, in the WA x NA cross, the hemizygous strains carrying the WA allele of *SAP185* or *SIT4*, showed greater consumption levels of ammonium (26% and 9.9%, respectively) compared to the strains with NA allele (Fig 4C and 4D). We did not observe differences for ammonium consumption in the WA x NA cross for *GTR1* and *SCH9* hemizygous, neither in the WA x WE cross for *SAP185* hemizygous (S4–S6 Tables), demonstrating that the impact of these genetic variants depends on the genetic background. However, the SA allele of *TOR2* had a negative effect (lower ammonium consumption) in two different hemizygous strains: SA x NA and SA x WA crosses, with a consumption difference of 7% and 8.7% compared to the strains carrying NA and WA alleles, respectively (Fig 4E). Altogether, our results demonstrate that allelic variants within the TORC1 pathway significantly impact ammonium consumption, which is depending of the genetic background analyzed.

### Allelic variants of non-wine origin for the TORC1 pathway are involved in higher amino acid consumption

**Fig 5:**
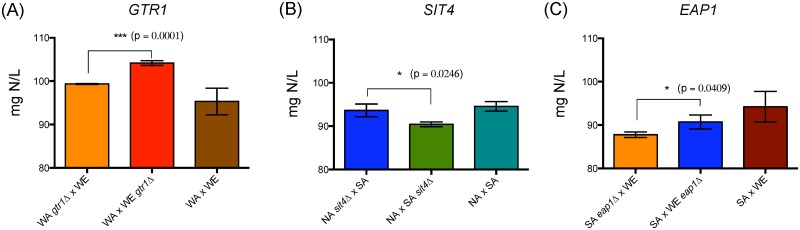
Reciprocal hemizygosity analysis for alleles affecting the amino acids consumption. Amino acids consumption for *GTR1* (A), *SIT4* (B), and *EAP1* (C) reciprocal hemizygous strains. Plotted values correspond to the average of three biological replicates, with their standard deviation represented by bars (mean ± SD). The asterisks represent different levels of significance between the phenotypes of the hemizygous strains (t-test; * p<0.05 and *** p<0.001).

**Fig 6:**
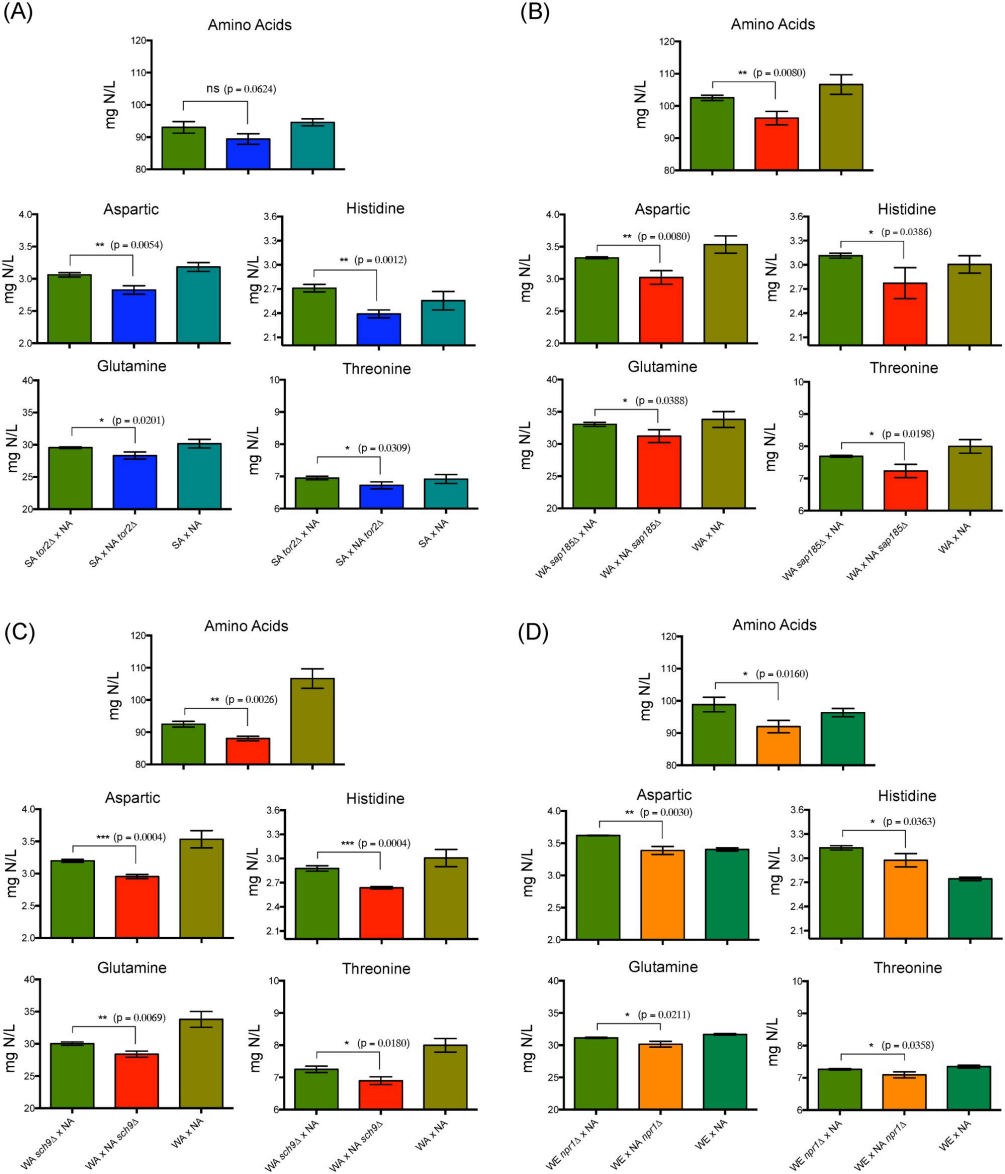
Allelic variants from the YPS128 (NA) strain showing higher consumption of amino acids. Amino acids consumption for *TOR2* (A), *SAP185* (B), *SCH9* (C) and *NPR1* (D) reciprocal hemizygous strains. Plotted values correspond to the average of three biological replicates, with their standard deviation represented by bars (mean ± SD). The asterisks represent different levels of significance between the phenotypes of the hemizygous strains (t-test; * p<0.05, ** p<0.01 and *** p<0.001).

Amino acids represent 60% of the total YAN available in synthetic must. Therefore, we analyzed the consumption profile for 17 amino acids in this set of genes. We observed significant differences in amino acid consumption in the hemizygous strains: *GTR1* (WA x WE), *SIT4* and *TOR2* (NA x SA), *SAP185* and *SCH9* (WA x NA), *NPR1* (WE x NA) and *EAP1* (SA x WE) (S4–S10 Tables). *GTR1* represents the single WA allele with a positive impact over the phenotype (higher amino acid consumption), with a higher consumption of aspartic acid, glutamic acid, serine, glutamine, threonine, alanine, tyrosine and valine; with an overall greater amino acid consumption (4.6%) compared to WE allele (Fig 5A). The SA allelic variant showed higher amino acid consumption in the hemizygous strains for *SIT4* and *EAP1* genes (Fig 5B and 5C). The SA allele of *SIT4* favored the consumption of aspartic acid, glutamic acid, serine, threonine, alanine, tyrosine, valine, isoleucine, phenylalanine and the total amount of amino acids, with a consumption difference of 3.4% compared to the NA allele (Fig 5B). The *EAP1* allele had the lowest impact on nitrogen consumption, with differences observed in only one of the crosses evaluated (SA x WE), and in five different amino acids: serine, glutamine, arginine, glycine, threonine and in the total amount of amino acids, which represents a differential consumption of 3.3% between the hemizygous strains (Fig 5C). Surprisingly, *SAP185*, *TOR2*, *SCH9* and *NPR1* allelic variants from the wild NA strains, isolated from a North American oak tree, presented higher consumption levels for amino acids, such as: aspartic acid, histidine, glutamine and threonine, with a differential consumption range between 2.4–12% and 4.8–6.9% in the total amount of amino acids (Fig 6).

Next, we evaluated the fermentation kinetics (15 mL fermentations) and the growth curves parameters (micro-cultivation) of each reciprocal hemizygous strain in MS300. The allelic variants of NA origin had an impact on the fermentative kinetic profiles and/or in the growth variables during micro-cultivation in MS300 (S5–S7 Figs). The hemizygous strain with NA allele for *SAP185* presented a higher fermentative efficiency (S5A Fig). This trend is also observed when we compared the growth variables obtained during micro-cultivation experiments, where the same hemizygous strain presented a lower lag time and generational time, also showing higher efficiency and maximum growth rate (S5B Fig). Therefore, the NA allele of this gene is beneficial for fermentation and growth kinetics in the assayed conditions. In this context, the NA allele of *TOR2* had a positive effect on the maximal CO_2_ production rate (S6 Fig), a parameter strongly dependent on nitrogen transporters and which correlates with nitrogen demand [35]. Finally, the hemizygous strain carrying the NA allele of *SCH9* presented a higher efficiency and maximum growth rate, also showing lower lag time and generational time in micro-cultivation experiments (S7 Fig). Overall, these results indicate that NA alleles of *SAP185*, *TOR2* and *SCH9* are beneficial for amino acid consumption during alcoholic fermentation and could be considered in genetic improvement programs.

In summary, we identified allelic variants within the TORC1 signaling pathway across four strains that significantly impact the regulatory mechanisms of nitrogen assimilation, resulting in nitrogen consumption differences between strains. Furthermore, similar nitrogen sources were affected by these allelic variants, which correspond to those described above in the PCA analysis.

### Relationship between allelic variants and nitrogen consumption

**Fig 7:**
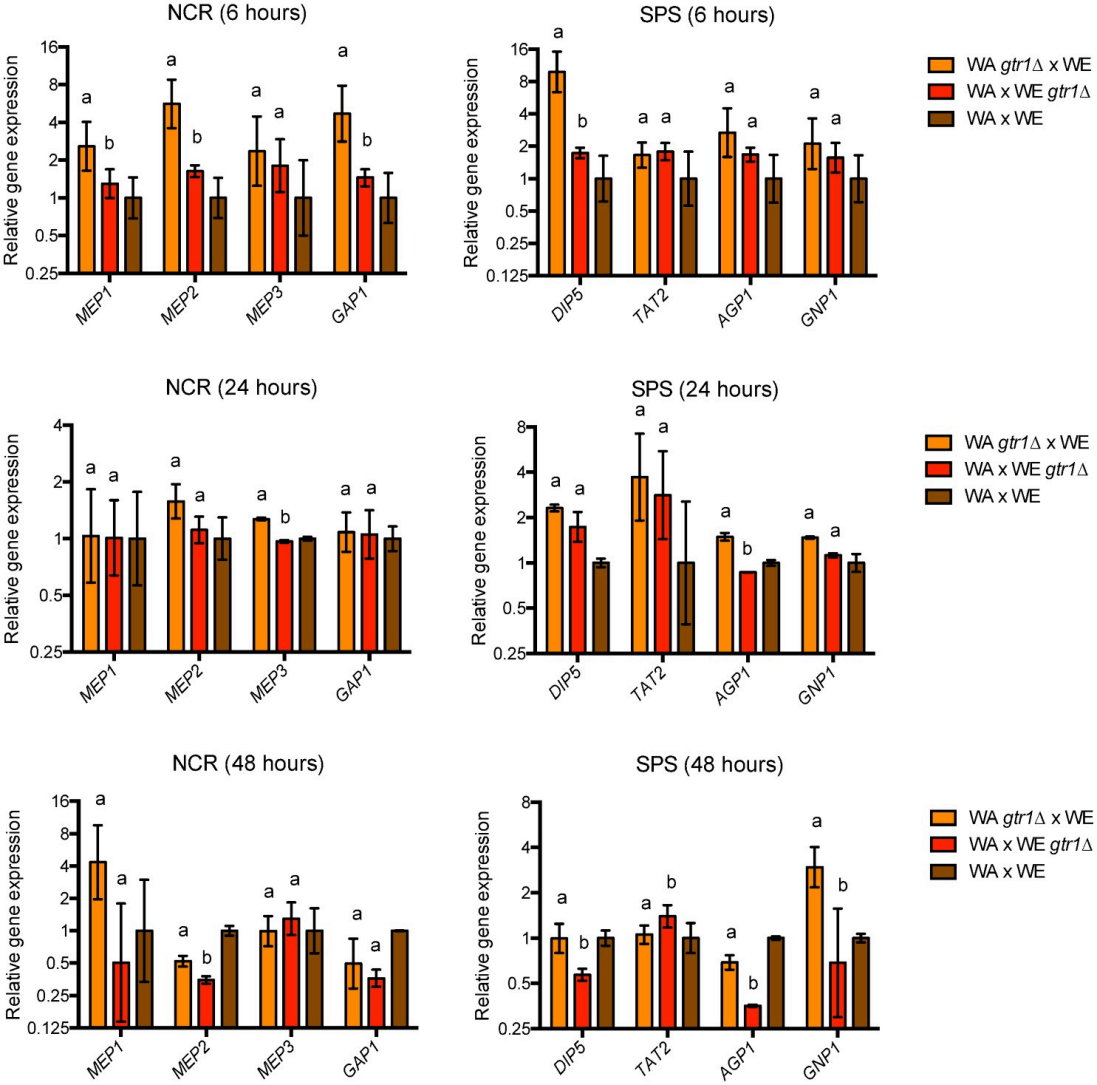
Relative gene expression for nitrogen transporters in the *GTR1* reciprocal hemizygous strains. Relative gene expression at 6, 24 and 48 hours of fermentation for nitrogen transporters controlled by NCR (*MEP1*, *MEP2*, *MEP3* and *GAP1*) and SPS (*DIP5*, *TAT2*, *AGP1* and *GNP1*) pathways, in the genetic background of *GTR1* hemizygous strains (WA x WE cross). Gene expression levels were normalized using three housekeeping genes and relative to the expression levels of the hybrid strain. Plotted values correspond to the average of two biological replicates, with their standard error represented by bars (mean ± error). Different letters represent significant statistical difference between the hemizygous strains (Mood test with p<0.05).

**Fig 8:**
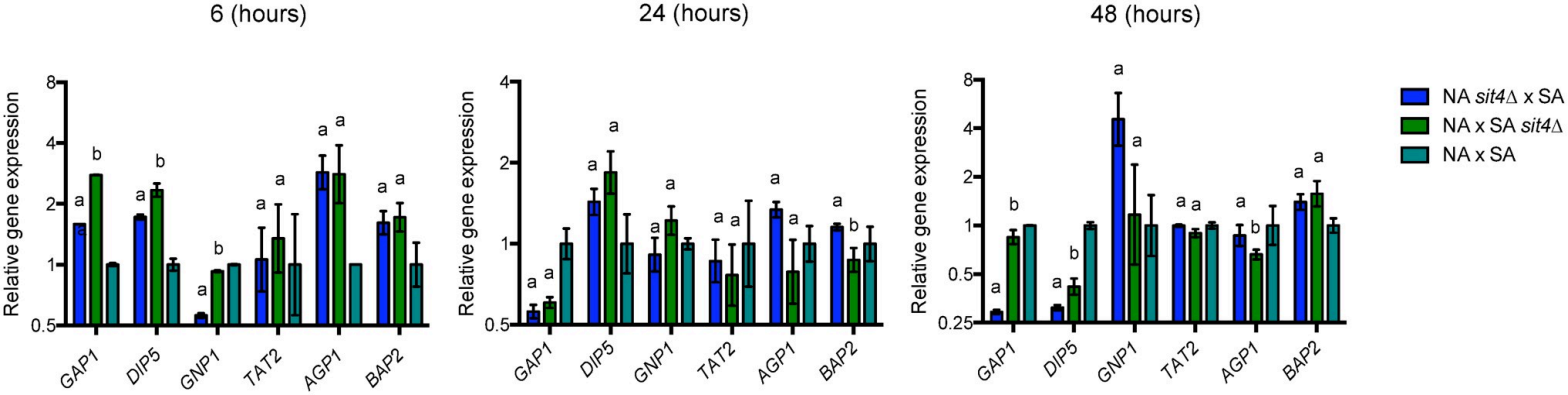
Relative gene expression for amino acid transporters in the *SIT4* reciprocal hemizygous strains. Relative gene expression at 6, 24 and 48 hours of fermentation for amino acid transporters (*GAP1*, *DIP5*, *GNP1*, *TAT2*, *AGP1* and *BAP2*), in the genetic background of *SIT4* hemizygous strains (NA x SA cross). Gene expression levels were normalized using three housekeeping genes and relative to the expression levels of the hybrid strain. Plotted values correspond to the average of two biological replicates, with their standard error represented by bars (mean ± error). Different letters represent significant statistical difference between the hemizygous strains (Mood test with p<0.05).

Previous studies have demonstrated that the TORC1 signaling pathway regulates the expression of nitrogen transporters [50–52]. Therefore, we estimated gene expression levels for genes encoding nitrogen transporters and then, we determined if the allelic variants identified affect the expression of those genes, which are downstream targets of the TORC1 pathway. For this, we evaluated the gene expression profile in the context of *GTR1* and *SIT4* reciprocal hemizygous strains (WA x WE and NA x SA crosses, respectively), since these genes are directly involved in the expression of nitrogen transporters. We selected *GTR1* hemizygous strains (WA x WE cross) due to the phenotypic difference observed in the hemizygous strain carrying the WE allele, which showed a superior ammonium consumption and reduced amino acid utilization respect to the strain with the WA allele (Figs 4A and 5A). In these hemizygous strains, we used qPCR to quantify the expression levels of transporters controlled by the NCR (*MEP1*, *MEP2*, *MEP3* and *GAP1*) and SPS (*DIP5*, *TAT2*, *AGP1* and *GNP1*) pathways (Fig 7), both controlled by TORC1. Similarly, we selected *SIT4* hemizygous strains (NA x SA cross) due to the reduced global amino acid consumption of the hemizygous strain carrying the NA allele compared to the strain with the SA allele (Fig 5B). In these hemizygous strains, we measured by qPCR the expression levels of amino acid transporters, such as: *GAP1*, *DIP5*, *GNP1*, *TAT2*, *AGP1* and *BAP2* (Fig 8). Overall, we choose three different stages of the fermentative process to compare gene expression, 6, 24 and 48 hours, corresponding to early and middle stages of the fermentative process, where the most significant changes in gene expression occurs [34].

In the case of *GTR1* gene, we observed a higher expression level of the nitrogen transporters (*MEP1* and *MEP2*) in the hemizygous strain with the WE allele (Fig 7), in agreement with higher ammonium consumption levels observed in this hemizygous strain. This hemizygous strain also showed a higher expression of all the transporters evaluated, except for *TAT2*, particularly 48 hours’ post-inoculation (Fig 7). On the other hand, when we compared the gene expression profile of *SIT4* reciprocal hemizygous strains, the hemizygous strain carrying the NA allele had higher expression of the transporters evaluated at 6 hours of fermentation (Fig 8). In this case, we did not find a correlation between gene expression levels for the amino acid transporters and the nitrogen consumption profiles. Altogether, these results suggest that *GTR1* and *SIT4* alleles could be affecting other mechanisms of nitrogen consumption, such as post-translational regulation.

## Discussion

One of the main challenges in yeast genetics is understanding the great diversity in nitrogen consumption profiles between strains [27]. Several studies have tried to elucidate the genetic basis of this phenotype, demonstrating that variations in nitrogen signaling pathways are responsible of differences in nitrogen consumption between strains during alcoholic fermentation [20,27]. In particular, it has been described that allelic variations in signaling pathways, such as TORC1 and MAPK, are the main factors responsible for phenotypic differences across strains [53,54]. In this sense, we determined if the allelic diversity in the TORC1 signaling pathway influence the nitrogen consumption differences between strains during the alcoholic fermentation.

The TORC1 signaling pathway controls the cell response to nitrogen quality and quantity [7]. Although it has been widely studied in laboratory conditions, little is known during fermentative process. In this context, TORC1 plays a key role in gene expression changes [29], controlling fermentation capacity in nitrogen starved cells [27]. Recently, a large phenotypic variation in the TORC1 activity has been reported in the same set of parental yeast strains that we used in this work [55]. For this, an upshift nitrogen experiment (proline to glutamine) was performed demonstrating that the SA strain has the greatest TORC1 activation compared to all the other strains, while the WE strain showed the weakest activity [55]. This variation in TORC1 activity coincide with the nitrogen consumption profiles of these strains, where the SA strain exhibits a preference for amino acid consumption and has a greater TORC1 activity. In contrast, the WE strain shows a preference for ammonium consumption with a low TORC1 activity, this is probably affecting the expression of NCR genes, increasing ammonium consumption. However, the molecular mechanism on how TORC1 signaling pathway generates nitrogen consumption variations in fermentative conditions is little understood. Here, we have shown how genetic variants in the TORC1 pathway underlie differences in nitrogen consumption between *S*. *cerevisiae* strains during the alcoholic fermentation. Consequently, our results demonstrated the great allelic diversity observed along the TORC1 signaling pathway, which generates variations in the downstream pathways that are subrogated to its activity. Thus, we have found seven different alleles related to the TORC1 signaling pathway (*GTR1*, *TOR2*, *SIT4*, *SAP185*, *EAP1*, *NPR1* and *SCH9*), which are affecting the ammonium and amino acid consumptions under fermentative conditions. Currently, we are further investigating the *GTR1* allele (Wine/European, WE) effect over the TORC1 activation, using a recently described method based on nitrogen upshift experiments and the luciferase reporter gene under *RPL26A* promoter control [55]. These exciting results, will allow us to associate the weakest TORC1 activation in the WE genetic background with the impaired function of its *GTR1* allele.

Initially, with the aim to evaluate how the TORC1 allelic variation impacts nitrogen consumption, we selected seven genes from a previous QTL mapping study [20], all of them participate in different stages of the TORC1 signaling pathway (Fig 2). Indeed, the allelic diversity present in these genes has a significant effect on nitrogen consumption during alcoholic fermentation. We observed statistically significant differences in the consumption of 12 out of 17 nitrogen sources evaluated (ammonium, arginine, serine, alanine, threonine, glutamic acid, isoleucine, valine, aspartic acid, phenylalanine, leucine and tyrosine), demonstrating the wide impact of the TORC1 pathway on nitrogen consumption. PCA analysis of these twelve nitrogen sources clearly shows a correlation according to the permeases utilized in the uptake: Mep1/Mep2/Mep3, Gap1, Gnp1, Agp1, Bap2 / Bap3, Tat1 / Tat2 and Dip5 [56–58], explaining over 66% of the observed variation. Likewise, various studies have demonstrated expression differences in genes encoding for nitrogen transporters [16,17,23], as one of the possible causes of diversity in nitrogen consumption. The expression of the transporters is partly regulated by the TORC1 signaling pathway [50], therefore, supporting our hypothesis that allelic variants in TORC1 could be impacting the regulatory mechanisms of these nitrogen transporters.

Ammonium consumption was affected by allelic variants in five genes: *GTR1*, *SCH9*, *TOR2*, *SAP185* and *SIT4*. However, differences in nitrogen consumption were observed across hemizygous strains depending on the ecological origin of the allele and the genetic background involved in the reciprocal cross, demonstrating a G x E interaction [32]. For example, the WA allele of *GTR1* has a negative effect (lower ammonium consumption) in the WE x WA cross. In contrast, the WA alleles of *TOR2*, *SAP185* and *SIT4* have a positive effect (higher ammonium consumption) in the SA x WA and WA x NA crosses (Fig 4), suggesting a complex gene-gene interaction [59–62]. In addition, this signaling pathway is conserved in eukaryotes [12], and any perturbations occurring on the pathway would result in compensatory perturbations, canceling or diminishing the effect of the first [62,63]. Alternative compensatory mechanisms could be a sign of epistasis, where one mutation has a negative effect on fitness in the presence of another mutation (non-additive interactions between mutations) [62], as a result of the linear hierarchy between signaling cascades [64]. This compensation over an allelic variant can also be observed in the WA x NA cross, while the NA allele of *SAP185* has a positive affect (higher amino acid consumption), the NA allele of *SIT4* has a negative effect (lower amino acid consumption) over this phenotype (Fig 4).

Previously, we identified allelic variants in four genes (*CPS1*, *ASI2*, *LYP1* and *ALP1*) that underlie nitrogen consumption differences. However, none of these allelic variants showed differences in ammonium consumption [20]. In this study, the WA allele of *GTR1* was the only one with a positive effect (higher amino acid consumption) in the three crosses evaluated. It is expected that the decrease in ammonium uptake will favor amino acid consumption in order to balance the central nitrogen metabolism, since all amino acids and ammonium end up as glutamine and glutamate [9]. Furthermore, the performance observed in both reciprocal hemizygous strains is consistent with the performance of the parental strains, because the WE strain consumes greater levels of ammonium compared to any of the other strains, while the WA strain is able to efficiently consume amino acids rather than ammonium [20], which is probably due to differences in nitrogen availability in the ecological niches from where the strains have been isolated [65]. The results for the WA allele of *GTR1*, suggest a favored interaction between EGO and the TORC1 complex, keeping the pathway activated, with downstream consequences in the expression of genes encoding nitrogen transporters regulated by SPS [11,52], and thus, increasing the amino acid consumption. In contrast, the results for the WE allele of *GTR1*, which consumed greater ammonium levels, suggest an inactivation of the TORC1 complex, independently of the intracellular amino acid concentration, activating the expression of NCR regulated genes and increasing ammonium consumption. Furthermore, a TORC1-independent role has been described for *GTR1*, where Gtr1p participates in the Gse complex that regulates the Gap1 cellular sorting from late endosome to plasmatic membrane. In this sense, different *GTR1* alleles could be regulating Gap1p levels in the plasmatic membrane, impacting intracellular amino acid levels and indirectly affecting TORC1 signaling [66,67]. Across the four strains, *GTR1* gene has two non-synonymous SNPs: R113S present in the WA strain and T150M present in the SA strain. These mutations are localized in the GTPase domain of the protein, likely affecting protein function. However, the SIFT prediction indicates that these mutations would be tolerated for protein function, and further analysis should be performed to determine whether these SNPs affect protein function. In consequence, the observed differences in nitrogen consumption could be due to mutations in the coding or regulatory regions, which could affect the GTPase activity of Gtr1p and the activation of the TORC1 complex, since its activation depends on the GTP-bound conformation of Gtr1p [68,69].

Amino acids represent approximately 60% of the YAN available in wine must [3]. We observed significant differences in the consumption of 11 amino acid sources (S4–S10 Tables), which represent 36% of the YAN available in wine must. Surprisingly, the NA alleles of four genes (*SAP185*, *TOR2*, *SCH9* and *NPR1*) have a positive effect (higher consumption) for aspartic acid, histidine, glutamine and threonine consumption, which represent 21% of the YAN available. *TOR2* participates in the TOR complex and, *SAP185* and *SCH9* are proximal outputs of the TORC1 complex [7], therefore, these genes could be modulating the activity of the pathway, whereas *NPR1* regulates nitrogen permeases and directly affects nitrogen consumption [70,71]. The NA strain is a wild isolate not adapted to wine must fermentation [65] and shows (globally) the lowest levels of amino acid consumption [20]. The large numbers of genes with a positive effect (higher amino acid consumption) is consistent with the complexity of quantitative traits [63]. Therefore, these allelic variants that augment amino acid consumption could be potentially used in genetic improvement programs, favoring amino acids consumption over ammonium in wine yeasts.

The differences observed in nitrogen consumption could be due to polymorphisms in the coding and/or regulatory regions [72]. Although, the TORC1 signaling pathway is highly conserved across species, from yeasts to humans [12], sequence analyses have revealed that despite its species-level conservation, all the genes evaluated (except for *SIT4*) contain non-synonymous polymorphisms in the coding regions. *SAP185* and *TOR2* are the most polymorphic genes studied; *TOR2* encodes a serine/threonine kinase and can participate in the two TOR complexes, TORC1 and TORC2. However, 90% of Tor2p is part of the TORC2 complex [73]. The results for the SA allele of *TOR2* suggests that Tor2p mainly participate in the TORC1 complex, which could affect the complex formation, generating a non-optima regulation of its targets genes and reducing the nitrogen consumption. This lower nitrogen consumption could impact the fermentative fitness, since the hemizygous strain containing the SA allele of *TOR2* showed a lower CO_2_ production rate (S6 Fig), trait strongly dependent on nitrogen transporters and correlates with the nitrogen demand [35]. Therefore, the SA allele of *TOR2* is detrimental for nitrogen consumption and fermentative fitness.

On the other hand, the WA allele of *SAP185* negatively impacts amino acid consumption, with consequences in growth and fermentative fitness (Fig 6 and S5 Fig). The SAPs proteins are considered the regulatory subunits of the phosphatase complex. *SAP185* null mutants present phenotypes such as defects in translation, a constitutive hyper-phosphorylating of the eIF2α, induction of *GCN4* translation, a higher expression of the NCR genes and a hyper-sensitivity to amino acid deprivation [74]. This is suggesting that the WA allele of *SAP185* could be generating a phenotype resembling the null mutant, increasing the dephosphorylated levels of its target proteins, including NCR genes and increasing ammonium consumption. The hemizygous strain carrying the WA allele of *SAP185* also showed higher consumption of tryptophan and leucine. The higher consumption of tryptophan could be a post-translational regulation of Tat2p, because the phosphatase complex dephosphorylates Npr1p and produces the stabilization of Tat2p in the plasmatic membrane [75]. Moreover, leucine is directly incorporated into proteins, and is not involved in the central nitrogen metabolism where its synthesis is in low levels [15]. Otherwise, *SIT4* is the only gene not containing non-synonymous SNPs between strains and, therefore, the phenotypic differences could be due to polymorphisms in the regulatory region. Thus, differences in gene expression for *SIT4* could be affecting the activity of the phosphatase complex. It has been demonstrated that Sit4p regulates the expression of genes under NCR and SPS control [51,52], in addition to post-translational regulation of ammonium and amino acid transporters via Npr1p [10,70].

In conclusion, our findings illustrate that although the TORC1 pathway is highly conserved in eukaryotes, it shows a high allelic diversity in yeast, specially for genes participating at different stages of the pathway. This generates variations in the nitrogen consumption profiles between strains during the fermentation. The allelic diversity could be affecting the activity of the TORC1 pathway and in consequence, the expression of its target genes. Finally, our results support the hypothesis of variations in nitrogen signaling pathways as the main cause of differences in nitrogen consumption in *S*. *cerevisiae* strains during the alcoholic fermentation.

## Supporting information

S1 Fig Chromosome localization of the candidate genes. Physical localization of the seven candidate genes: *SIT4* (orange rectangle), *SCH9* (blue rectangle), *SAP185* (red rectangle), *TOR2* (light blue rectangle), *EAP1* (pink rectangle), *GTR1* (green rectangle) and *NPR1* (purple rectangle). Centromere (black circle) is also indicated.
(TIF)

S2 Fig Selection of parental strains for posterior reciprocal hemizygosity analysis. Nitrogen consumption levels in segregant strains carrying WE, NA, SA or WA allele for Chr X QTL: 235087 bp (*SAP185*) (A); Chr XI QTL: 54205 bp (*TOR2* / *EAP1*) (B); Chr XIV QTL: 281995 bp (*NPR1*) (C); Chr IV QTL: 371305 bp (*SIT4*) (D); Chr VIII QTL: 505776 bp (*SCH9*) (E); and, Chr XIII QTL: 22769 bp (*GTR1*) (F).
(TIF)

S3 Fig Spearman correlation for amino acids from group 1. Spearman correlation for: serine versus glutamine (A), threonine (B) and alanine (C); glutamine versus threonine (D), alanine (E), arginine (F) and isoleucine (G); arginine versus threonine (H), alanine (I) and isoleucine (J); threonine versus alanine (K) and isoleucine (L); and alanine versus isoleucine (M).
(TIF)

S4 Fig Spearman correlation for amino acids from group 2. Spearman correlation for: aspartic versus tyrosine (A), valine (B), leucine (C) and phenylalanine (D); tyrosine versus valine (E), leucine (F) and phenylalanine (G); valine versus leucine (H) and phenylalanine (I); leucine versus phenylalanine (J).
(TIF)

S5 Fig Fermentation rate and growth parameters in the reciprocal hemizygous strains for *SAP185* gene. Fermentative performance (WA x NA cross) was evaluated by CO_2_ loss and extracting the kinetics parameters from the curves (A). The growth performance was evaluated by growth curves and extracting the kinetics parameters (B). Plotted values correspond to the average of three biological replicates, with their standard deviation represented by bars (mean ± SD). The asterisk represents a statistically significant different between the phenotypes of the hemizygous strains (t-test; * p<0.05).
(TIF)

S6 Fig Fermentation performance in the reciprocal hemizygous strains for *TOR2* gene. CO_2_ loss curves and their extracted kinetic parameters for the hemizygous strains (SA x NA cross). Plotted values correspond to the average of three biological replicates, with their standard deviation represented by bars (mean ± SD). The double asterisks represent a statistically significant different between the phenotypes of the hemizygous strains (t-test; ** p<0.01).
(TIF)

S7 Fig Growth performance of the reciprocal hemizygous strains for *SCH9* gene. Growth curves and its extracted kinetic parameters for the hemizygous strains (WA x NA cross). Plotted values correspond to the average of three biological replicates, with their standard deviation represented by bars (mean ± SD). The asterisks represent different levels of significance between the phenotypes of the hemizygous strains (t-test; * p<0.05, ** p<0.01).
(TIF)

S1 Table List of strains used in this study. (PDF)

S2 Table List of primers used in this study. (PDF)

S3 Table SNPs present in the seven candidate genes. (PDF)

S4 Table Nitrogen consumption (mgN/L) for *GTR1* reciprocal hemizygous strains. (PDF)

S5 Table Nitrogen consumption (mgN/L) for *SCH9* reciprocal hemizygous strains. (PDF)

S6 Table Nitrogen consumption (mgN/L) for *SAP185* reciprocal hemizygous strains. (PDF)

S7 Table Nitrogen consumption (mgN/L) for *SIT4* reciprocal hemizygous strains. (PDF)

S8 Table Nitrogen consumption (mgN/L) for *TOR2* reciprocal hemizygous strains. (PDF)

S9 Table Nitrogen consumption (mgN/L) for *NPR1* reciprocal hemizygous strains. (PDF)

S10 Table Nitrogen consumption (mgN/L) for *EAP1* reciprocal hemizygous strains. (PDF)

S11 Table Nitrogen consumption (mgN/L) for hybrids strains. (PDF)
