# Supplementary figures and images for: Genetic variants of TORC1 signaling pathway affect nitrogen consumption in Saccharomyces cerevisiae during alcoholic fermentation
Source: PLoS One. 2019 Jul 26;14(7):e0220515. doi: 10.1371/journal.pone.0220515 (PMC6660096; doi:10.1371/journal.pone.0220515)

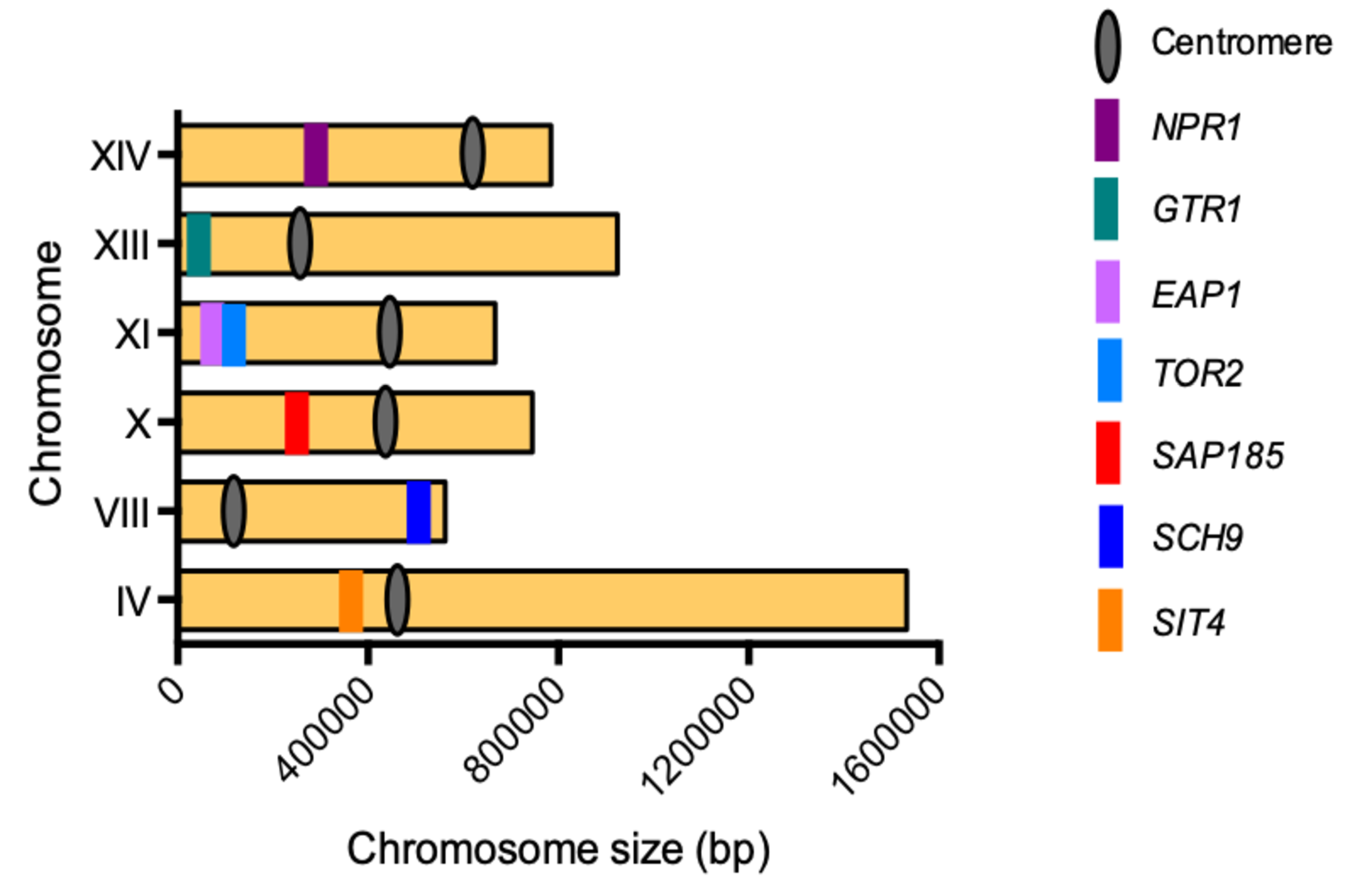

Supplement: S1 Fig — Physical localization of the seven candidate genes: SIT4 (orange rectangle), SCH9 (blue rectangle), SAP185 (red rectangle), TOR2 (light blue rectangle), EAP1 (pink rectangle), GTR1 (green rectangle) and NPR1 (purple rectangle). Centromere (black circle) is also indicated. (TIF) [file pone.0220515.s001.tif]

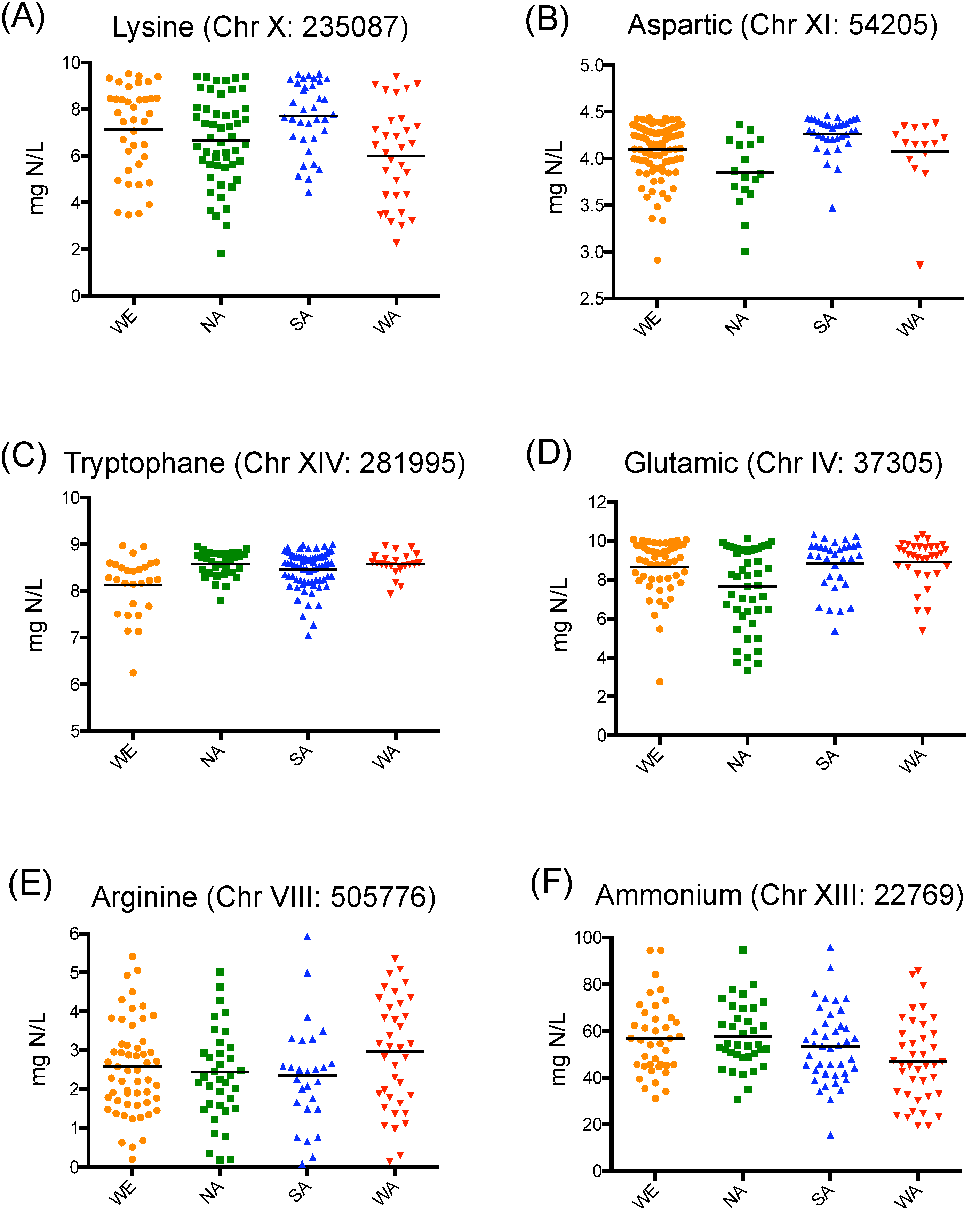

Supplement: S2 Fig — Nitrogen consumption levels in segregant strains carrying WE, NA, SA or WA allele for Chr X QTL: 235087 bp (SAP185) (A); Chr XI QTL: 54205 bp (TOR2 / EAP1) (B); Chr XIV QTL: 281995 bp (NPR1) (C); Chr IV QTL: 371305 bp (SIT4) (D); Chr VIII QTL: 505776 bp (SCH9) (E); and, Chr XIII QTL: 22769 bp (GTR1) (F). (TIF) [file pone.0220515.s002.tif]

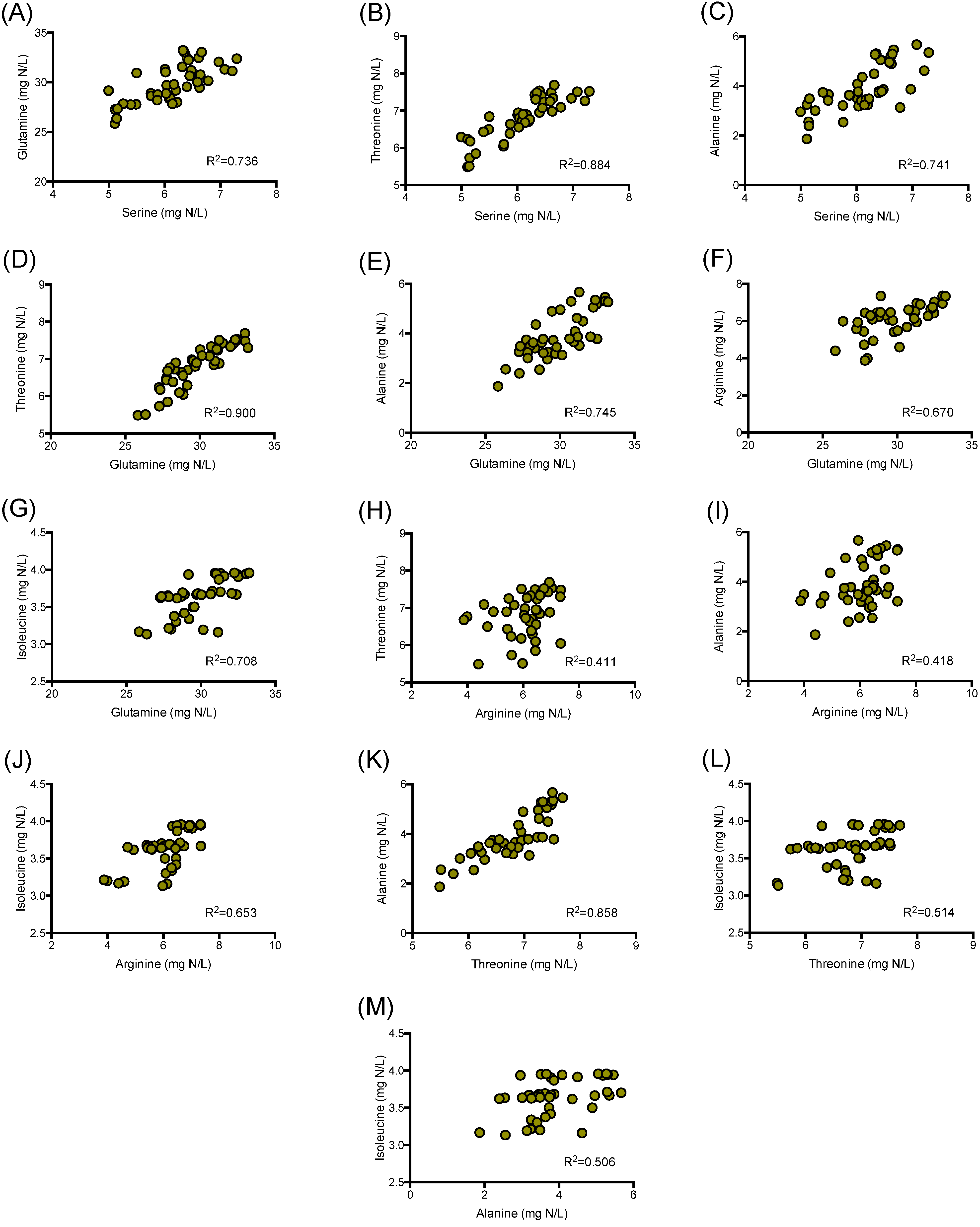

Supplement: S3 Fig — Spearman correlation for: serine versus glutamine (A), threonine (B) and alanine (C); glutamine versus threonine (D), alanine (E), arginine (F) and isoleucine (G); arginine versus threonine (H), alanine (I) and isoleucine (J); threonine versus alanine (K) and isoleucine (L); and alanine versus isoleucine (M). (TIF) [file pone.0220515.s003.tif]

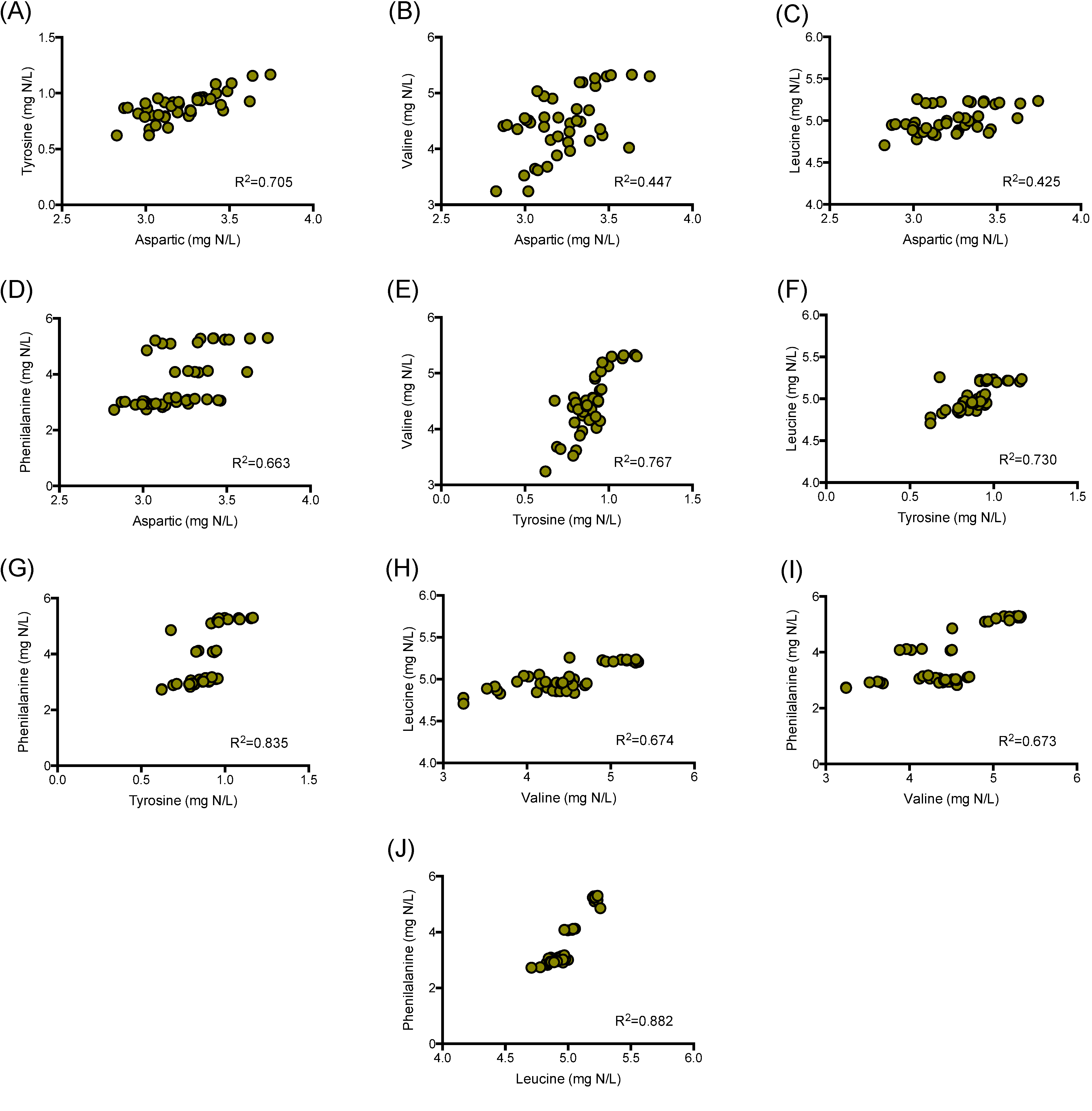

Supplement: S4 Fig — Spearman correlation for: aspartic versus tyrosine (A), valine (B), leucine (C) and phenylalanine (D); tyrosine versus valine (E), leucine (F) and phenylalanine (G); valine versus leucine (H) and phenylalanine (I); leucine versus phenylalanine (J). (TIF) [file pone.0220515.s004.tif]

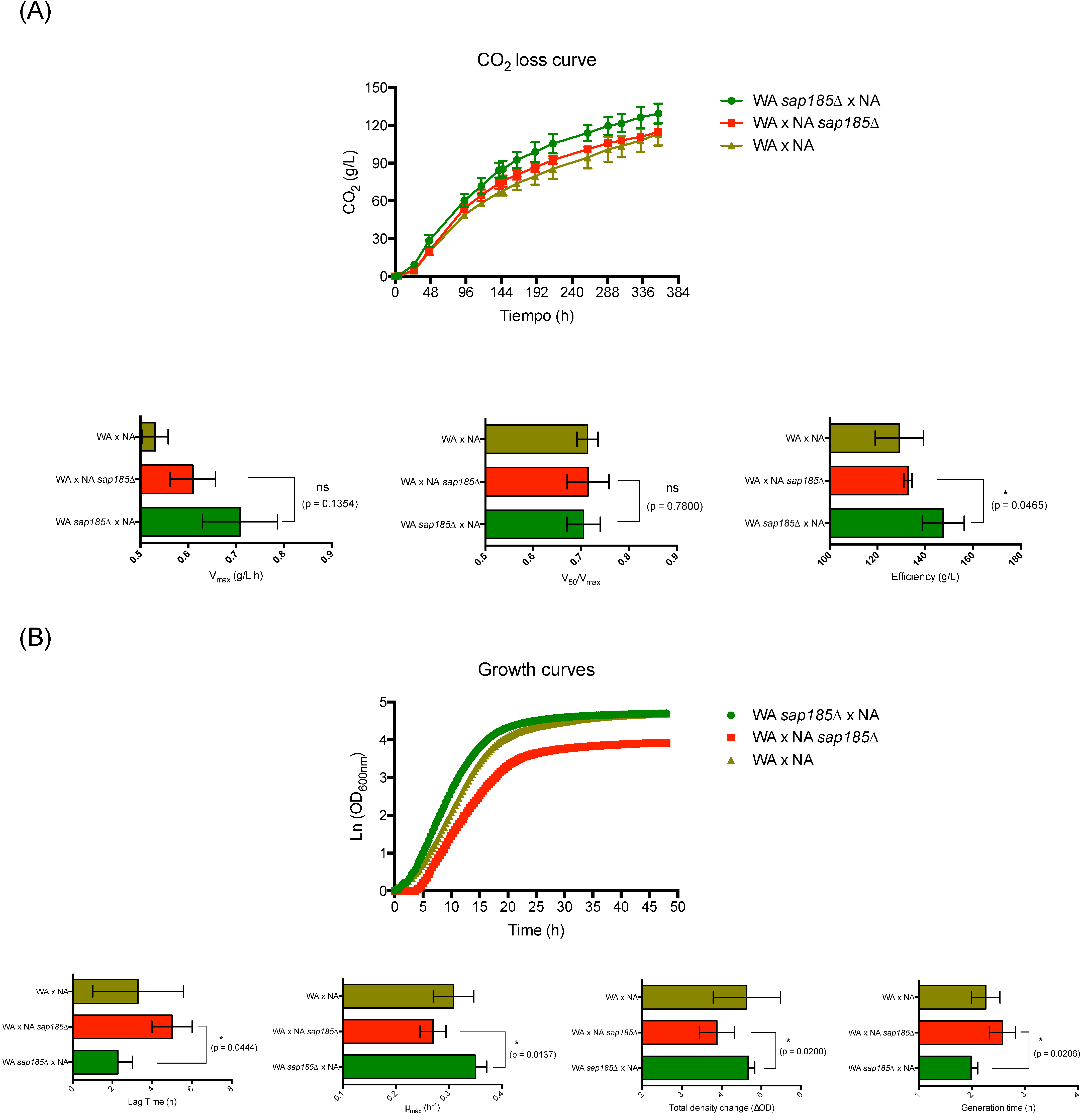

Supplement: S5 Fig — Fermentative performance (WA x NA cross) was evaluated by CO2 loss and extracting the kinetics parameters from the curves (A). The growth performance was evaluated by growth curves and extracting the kinetics parameters (B). Plotted values correspond to the average of three biological replicates, with their standard deviation represented by bars (mean ± SD). The asterisk represents a statistically significant different between the phenotypes of the hemizygous strains (t-test; * p<0.05). (TIF) [file pone.0220515.s005.tif]

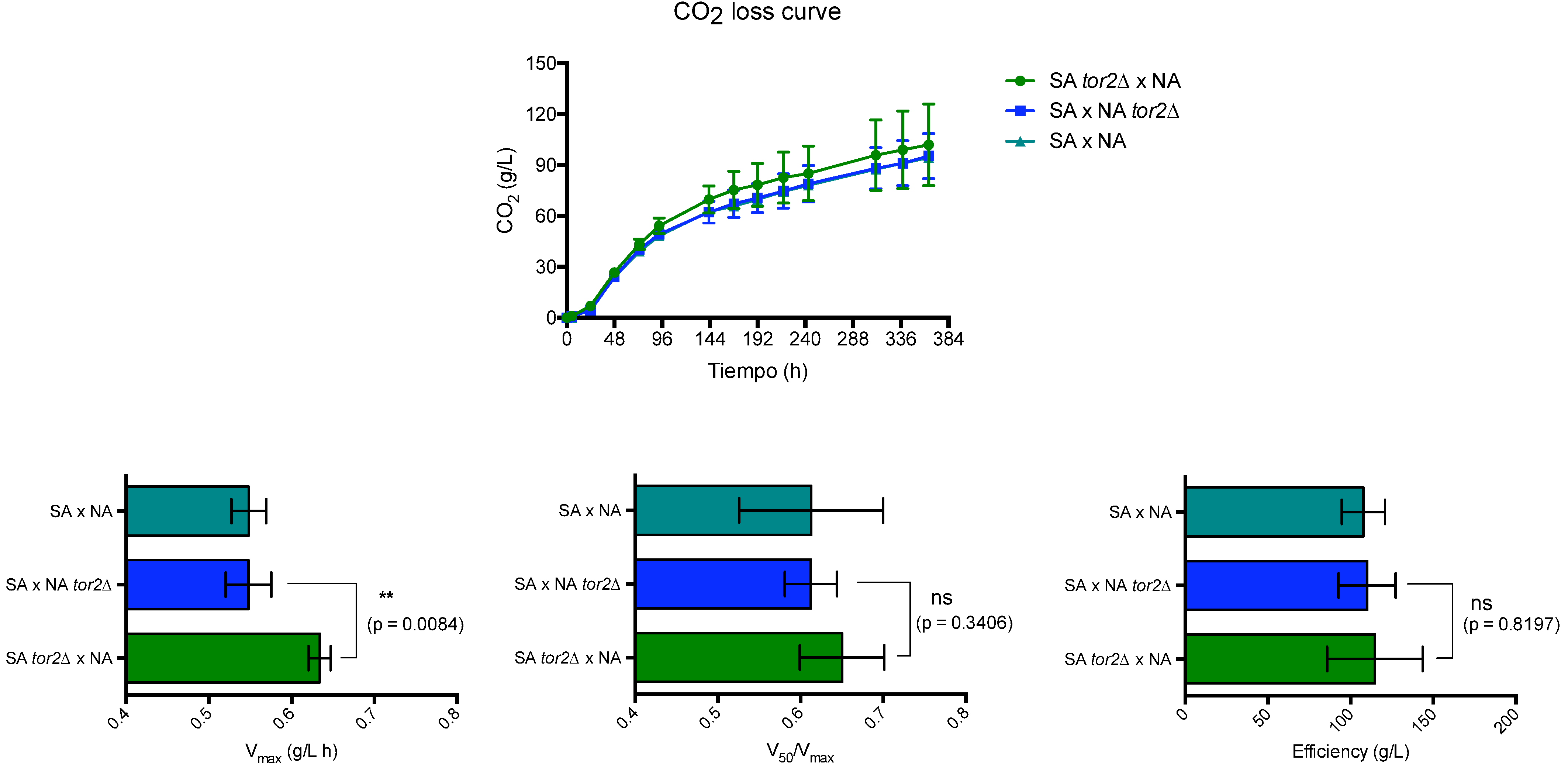

Supplement: S6 Fig — CO2 loss curves and their extracted kinetic parameters for the hemizygous strains (SA x NA cross). Plotted values correspond to the average of three biological replicates, with their standard deviation represented by bars (mean ± SD). The double asterisks represent a statistically significant different between the phenotypes of the hemizygous strains (t-test; ** p<0.01). (TIF) [file pone.0220515.s006.tif]

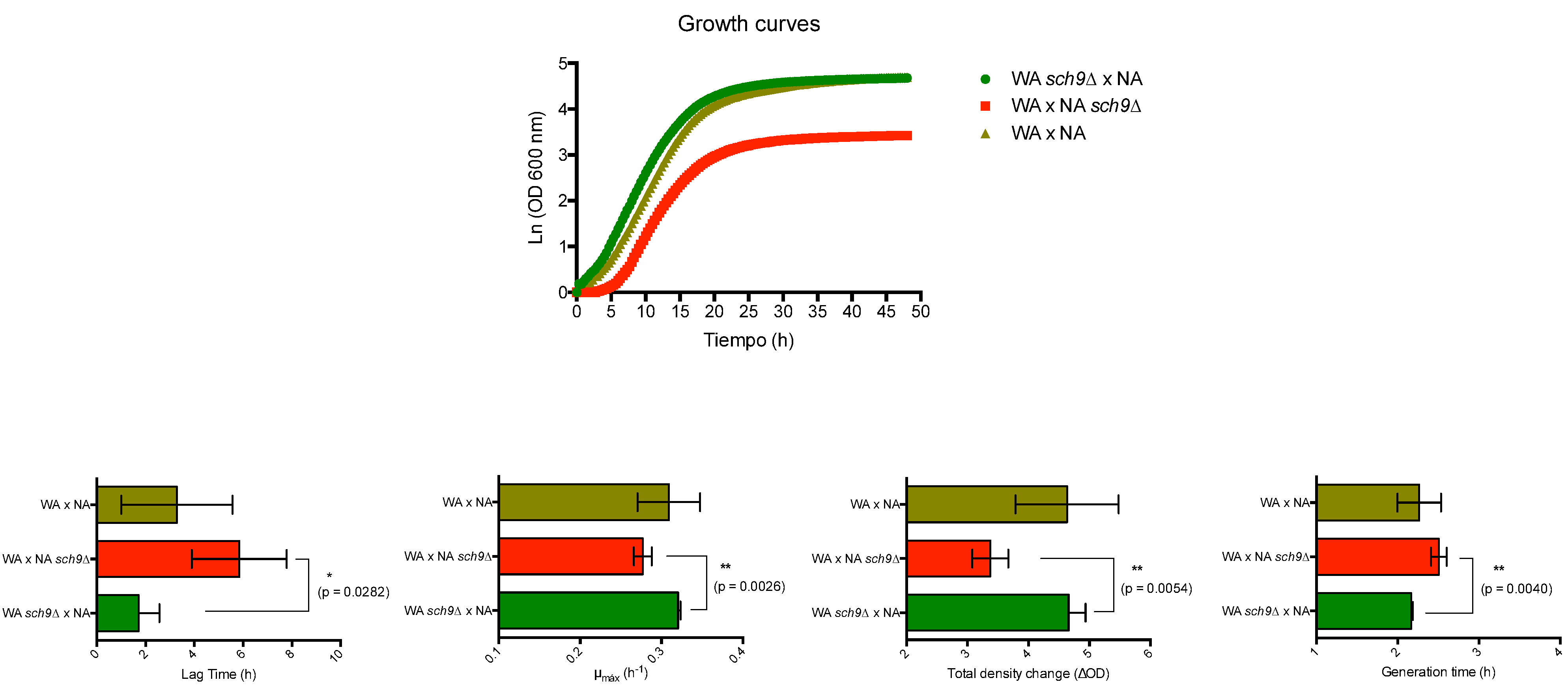

Supplement: S7 Fig — Growth curves and its extracted kinetic parameters for the hemizygous strains (WA x NA cross). Plotted values correspond to the average of three biological replicates, with their standard deviation represented by bars (mean ± SD). The asterisks represent different levels of significance between the phenotypes of the hemizygous strains (t-test; * p<0.05, ** p<0.01). (TIF) [file pone.0220515.s007.tif]
