# Supplementary material for: Genetic variants of TORC1 signaling pathway affect nitrogen consumption in Saccharomyces cerevisiae during alcoholic fermentation
Source: PLoS One. 2019 Jul 26;14(7):e0220515. doi: 10.1371/journal.pone.0220515 (PMC6660096; doi:10.1371/journal.pone.0220515)
Supplement: S1 Table — (PDF) [file pone.0220515.s008.pdf]

S1 Table. List of strains used in this study.

Strain	Genotype	Reference
DBVPG6765 (WE)	*MATa*, *ho::HygMX*, *ura3::KanMX*	[32]
	MATα, *ho::NatMX*, *ura3::KanMX*	[32]
DBVPG6044 (WA)	*MATa*, *ho::HygMX*, *ura3::KanMX*	[32]
	MATα, *ho::NatMX*, *ura3::KanMX*	[32]
YPS128 (NA)	*MATa*, *ho::HygMX*, *ura3::KanMX*	[32]
	MATα, *ho::NatMX*, *ura3::KanMX*	[32]
Y12 (SA)	*MATa*, *ho::HygMX*, *ura3::KanMX*	[32]
	MATα, *ho::NatMX*, *ura3::KanMX*	[32]
NA *sit4*Δ x WE	*sit4* (NA)::*URA3*, *ho::HygMX*, *ho::NatMX*, *ura3::KanMX*	This study
NA x WE *sit4*Δ	*sit4* (WE)::*URA3*, *ho::HygMX*, *ho::NatMX*, *ura3::KanMX*	This study
NA *sit4*Δ x WA	*sit4* (NA)::*URA3*, *ho::HygMX*, *ho::NatMX*, *ura3::KanMX*	This study
NA x WA *sit4*Δ	*sit4* (WA)::*URA3*, *ho::HygMX*, *ho::NatMX*, *ura3::KanMX*	This study
NA *sit4*Δ x SA	*sit4* (NA)::*URA3*, *ho::HygMX*, *ho::NatMX*, *ura3::KanMX*	This study
NA x SA *sit4*Δ	*sit4* (SA)::*URA3*, *ho::HygMX*, *ho::NatMX*, *ura3::KanMX*	This study
WA *sap185*Δ x WE	*sap185* (WA)::*URA3*, *ho::HygMX*, *ho::NatMX*, *ura3::KanMX*	This study
WA x WE *sap185*Δ	*sap185* (WE)::*URA3*, *ho::HygMX*, *ho::NatMX*, *ura3::KanMX*	This study
WA *sap185*Δ x NA	*sap185* (WA)::*URA3*, *ho::HygMX*, *ho::NatMX*, *ura3::KanMX*	This study
WA x NA *sap185*Δ	*sap185* (NA)::*URA3*, *ho::HygMX*, *ho::NatMX*, *ura3::KanMX*	This study
WA *sap185*Δ x SA	sap185 (WA)::*URA3*, *ho::HygMX*, *ho::NatMX*, *ura3::KanMX*	This study
WA x SA *sap185*Δ	*sap185* (SA)::*URA3*, *ho::HygMX*, *ho::NatMX*, *ura3::KanMX*	This study
SA *eap1*Δ x WE	eap1 (SA)::*URA3*, *ho::HygMX*, *ho::NatMX*, *ura3::KanMX*	This study
SA x WE *eap1*Δ	eap1 (WE)::*URA3*, *ho::HygMX*, *ho::NatMX*, *ura3::KanMX*	This study
SA *eap1*Δ x WA	eap1 (SA)::*URA3*, *ho::HygMX*, *ho::NatMX*, *ura3::KanMX*	This study
SA x WA *eap1*Δ	eap1 (WA)::*URA3*, *ho::HygMX*, *ho::NatMX*, *ura3::KanMX*	This study
SA *eap1*Δ x NA	eap1 (SA)::*URA3*, *ho::HygMX*, *ho::NatMX*, *ura3::KanMX*	This study
SA x NA *eap1*Δ	eap1 (NA)::*URA3*, *ho::HygMX*, *ho::NatMX*, *ura3::KanMX*	This study
WA *gtr1*Δ x WE	gtr1 (WA)::*URA3*, *ho::HygMX*, *ho::NatMX*, *ura3::KanMX*	This study

WA x WE *gtr1*Δ	gtr1 (WE)::*URA3*, *ho::HygMX*, *ho::NatMX*, *ura3::KanMX*	This study
WA *gtr1*Δ x NA	gtr1 (WA)::*URA3*, *ho::HygMX*, *ho::NatMX*, *ura3::KanMX*	This study
WA x NA *gtr1*Δ	gtr1 (NA)::*URA3*, *ho::HygMX*, *ho::NatMX*, *ura3::KanMX*	This study
WA *gtr1*Δ x SA	gtr1 (WA)::*URA3*, *ho::HygMX*, *ho::NatMX*, *ura3::KanMX*	This study
WA x SA *gtr1*Δ	gtr1 (SA)::*URA3*, *ho::HygMX*, *ho::NatMX*, *ura3::KanMX*	This study
WA *sch9*Δ x WE	sch9 (WA)::*URA3*, *ho::HygMX*, *ho::NatMX*, *ura3::KanMX*	This study
WA x WE *sch9*Δ	sch9 (WE)::*URA3*, *ho::HygMX*, *ho::NatMX*, *ura3::KanMX*	This study
WA *sch9*Δ x NA	sch9 (WA)::*URA3*, *ho::HygMX*, *ho::NatMX*, *ura3::KanMX*	This study
WA x NA *sch9*Δ	sch9 (NA)::*URA3*, *ho::HygMX*, *ho::NatMX*, *ura3::KanMX*	This study
WA *sch9*Δ x SA	sch9 (WA)::*URA3*, *ho::HygMX*, *ho::NatMX*, *ura3::KanMX*	This study
WA x SA *sch9*Δ	sch9 (SA)::*URA3*, *ho::HygMX*, *ho::NatMX*, *ura3::KanMX*	This study
WE *npr1*Δ x WA	npr1 (WE)::*URA3*, *ho::HygMX*, *ho::NatMX*, *ura3::KanMX*	This study
WE x WA *npr1*Δ	npr1 (WA)::*URA3*, *ho::HygMX*, *ho::NatMX*, *ura3::KanMX*	This study
WE *npr1*Δ x NA	npr1 (WE)::*URA3*, *ho::HygMX*, *ho::NatMX*, *ura3::KanMX*	This study
WE x NA *npr1*Δ	npr1 (NA)::*URA3*, *ho::HygMX*, *ho::NatMX*, *ura3::KanMX*	This study
WE *npr1*Δ x SA	npr1 (WE)::*URA3*, *ho::HygMX*, *ho::NatMX*, *ura3::KanMX*	This study
WE x SA *npr1*Δ	npr1 (SA)::*URA3*, *ho::HygMX*, *ho::NatMX*, *ura3::KanMX*	This study
WE x WA	ho::HygMX, ho::NatMX, ura3::KanMX	This study
WE x NA	ho::HygMX, ho::NatMX, ura3::KanMX	This study
WE x SA	ho::HygMX, ho::NatMX, ura3::KanMX	This study
WA x NA	ho::HygMX, ho::NatMX, ura3::KanMX	This study
WA x SA	ho::HygMX, ho::NatMX, ura3::KanMX	This study
NA x SA	ho::HygMX, ho::NatMX, ura3::KanMX	This study
