# Supplementary material for: Genetic variants of TORC1 signaling pathway affect nitrogen consumption in Saccharomyces cerevisiae during alcoholic fermentation
Source: PLoS One. 2019 Jul 26;14(7):e0220515. doi: 10.1371/journal.pone.0220515 (PMC6660096; doi:10.1371/journal.pone.0220515)
Supplement: S2 Table — (PDF) [file pone.0220515.s009.pdf]

S2 Table. **List of primers used in this study.**

Gene	Description	Name	Oligonucleotide sequence (5'-3')	Reference
SIT4	Disruption cassette*	SIT4_FW	CTT CAG TGG AAA CCC AGT GAT GCT CGC ACA CAA CGT AAT GGA GCA TTG AAG AGC TAC AGA AGC TTT TCA ATT CAT CAT	This study
		SIT4_RV	TGC TTG TTG TGT ATC GTA TCG TAG CAA ATG GCG TAA TGA AAA AGT TTC TGA TTT TTG TAG AGC TTT TTC TTT CCA ATT	This study
	PCR verification	A1_SIT4	TTT CGC TCT CGC TCT CTT TC	This study
		A4_SIT4	TCG GCA CGA TGA ACG TAA TA	This study
SAP185	Disruption cassette*	SAP185_FW	CAT AGG AGA CGA AAT CAT GGT CAT GAT CGA TGC CAT GCT TGC GTG GAT AGC CGA CCT AGA AGC TTT TCA ATT CAT CAT	This study
		SAP185_RV	GAC CAA CCA ACA GCA GAA AGC AAA TTA TAA AGG GGT AAG AAG GAC AAT GGT GAC GCA AGT AGC TTT TTC TTT CCA ATT	This study
	PCR verification	A1_SAP185	TTG ATC ATG CTG CGT ACC TG	This study
		A4_SAP185	AGC AAT GGG GAA CGT ACA AC	This study
EAP1	Disruption cassette*	EAP1_FW	GAA TGG GAC CTG CTT GGA TAA TGA CGA CAT CAA TAA AGA TCC CAA CCT TAA AAT AGA TGG AGC TTT TCA ATT CAT CAT	This study
		EAP1_RV	AAC ATT ATA TCG GTT GGT GTC CAT TCT GGT AGT TTT AAT GTA TTG AAA ATC ACT TAG TTG AGC TTT TTC TTT CCA ATT	This study
	PCR verification	A1_EAP1	GAA AAA GGC TTA CTG CAC AT	This study
		A4_EAP1	TAA CGG GGA ACG ATA TAA GA	This study
TOR2	Disruption cassette*	TOR2_FW	CTG ACA TAT ATG GCA GCA ATT AAA ATT GGA AGA AAT GTA ATG ACA GTA GGA AAG ACC AAT AGC TTT TCA ATT CAT CAT	This study
		TOR2_RV	ATG TTT TCG CCT TTG ATA TTT ACT AGT CGA AGG AAC TTT TTT CGC AGT TAG TAA CGT CAC AGC TTT TTC TTT CCA ATT	This study
	PCR verification	A1_TOR2	TTC GAC GGA ATC GTG GTA AT	This study
		A4_TOR2	GAG ATG CGT AAC GAG CGA GT	This study
GTR1	Disruption cassette*	GTR1_FW	CTA TAA CAG CAC CAA CGT GCG TAA TAC TGA TTG CTG TCA TTT CTT GGC ATG TTT TCT TGG AGC TTT TCA ATT CAA TTC ATC AT	This study
		GTR1_RV	TTC TTA TGC CCC GCA AAC ACT CAA TTG CCG AAT GTT TCG TCT ACT CAC CTC AGT CAT TGG AGC TTT TTC TTT CCA ATT	This study
	PCR verification	A1_GTR1	AAC TGC ACC GAT CTC ACT GA	This study
		A4_GTR1	TAA CAC ACA CAC ACA CGC ACA AA	This study
SCH9	Disruption cassette*	SCH9_FW	GAT CCT TAA AGG CTT ACT TAT TCA CAT TAC GGG TCC AAT ATA ACA TAG ATT GTT GTG CTC AGC TTT TCA ATT CAT CAT	This study
		SCH9_RV	GAG GGG TAA ATA TGT CAA AGT ACA ATG GAA TGG TGA GGT ATA AGC AGT GAT TAT GAG ACG *AGC TTT TTC TTT CCA ATT*	This study
	PCR verification	A1_SCH9	TCG TTA CCC TCG GTA AAA CC	This study
		A4_SCH9	ATT TCC GAA CCG ACT TTG G	This study
NPR1	Disruption cassette*	NPR1_FW	ACA TCT CAT TCT CAA TCT GAC TGA TCA TCT ATT ACG GAA TTT TTT CGT TAT CAA	This study

			TGA CGC AGC TTT TCA ATT CAA TTC ATC AT	
		NPR1_RV	ATT GAT TAT TTT GCT TTT TCT TTT TCT TTT CTA GGC CTG CAA TAT GTG CTT CAC TTT GAT AGC TTT TTC TTT CCA ATT	This study
	PCR verification	A1_NPR1	TCT TTA GAC AGC AAT GCC GC	This study
		A4_NPR1	AGT GGC GCG ATA TTT TAA CGA	This study
URA3	PCR verification	S8	CCT CTA GGT TCC TTT GTT ACT TCT	[41]
URA3	PCR verification	S5	CCT TTT GAT GTT AGC AGA ATT GTC	[41]
ACT1	Primer real-time PCR	ACT1-F	TTG GCC GGT AGA GAT TTG AC	[19]
		ACT1-R	CCC AAA ACA GAA GGT GGA A	[19]
UBC6	Primer real-time PCR	UBC6-F	GATACTTGGAATCCTGGCTGGTCTGTCTC	[47]
		UBC6-R	AAAGGGTCTTCTGTTTCATCACCTGTATTTGC	[47]
RPN2	Primer real-time PCR	RPN2-F	GCGGATACAGGCACATTGGATACC	[47]
		RPN2-R	TGTTGCTACCTTCTCTACCTCCTTACC	[47]
MEP1	Primer real-time PCR	MEP1-F	CCT GAG CTC GCG TAT GCA	[24]
		MEP1-R	GGC GCC AGC GAT AAT ACT TAA	[24]
MEP2	Primer real-time PCR	MEP2-F	GGT ATC ATC GCT GGC CTA GTG	[24]
		MEP2-R	ACA ACG GCT GAC CAG ATT GG	[24]
MEP3	Primer real-time PCR	MEP3-F	GCC GGT GTG GTG GGA TT	[24]
		MEP3-R	TTG TGC CGT CCA TTC CAA T	[24]
GAP1	Primer real-time PCR	GAP1-F	CTG TGG ATG CTG CTG CTT CA	[24]
		GAP1-R	CAA CAC TTG GCA AAC CCT TGA	[24]
DIP5	Primer real-time PCR	DIP5-F	TGG TTG CCA TTC AAA ACT CA	This study
		DIP5-R	CGA AAA CCA AGA CAC AAG CA	This study
TAT2	Primer real-time PCR	TAT2-F	CTG GCC ACG TGC ATT GTC T	[26]
		TAT2-R	GCC TTC ATC GCC AGT CTA AAT C	[26]
AGP1	Primer real-time PCR	AGP1-F	CGC CAT ATG TCA TTG CTG TTG	[21]
		AGP-R	CAT GGA CAG CAC GGA AAG TAG A	[21]
GNP1	Primer real-time PCR	GNP1-F	TCG TGT GGT TCC TCA TTT CAT AA	[26]
		GNP1-R	CCG TTA GCA ACG GAA AGA ACA	[26]
BAP2	Primer real-time PCR	BAP2-F	TGG TTG GCC TTT TAC TTC GGA	[25]
		BAP2-R	TTC GTC CTC TTG TCT CAT TAG	[25]

*Underlining indicates homology to *URA3* cassette. The remaining sequences of the primers are homologous to the flanking region of the target genes.
