# Supplementary material for: Genetic variants of TORC1 signaling pathway affect nitrogen consumption in Saccharomyces cerevisiae during alcoholic fermentation
Source: PLoS One. 2019 Jul 26;14(7):e0220515. doi: 10.1371/journal.pone.0220515 (PMC6660096; doi:10.1371/journal.pone.0220515)
Supplement: S3 Table — (PDF) [file pone.0220515.s010.pdf]

S3 Table. SNPs present in the seven candidate genes.

Gene	Regulatory region	Coding region	
		Synonymous SNP	Non-synonymous SNP
SAP185	8	26	18
EAP1	6	6	2
TOR2	7	38	7
NPR1	12	12	2
SIT4	7	1	0
SCH9	9	16	2
GTR1	8	2	2
