# Supplementary material for: Genetic variants of TORC1 signaling pathway affect nitrogen consumption in Saccharomyces cerevisiae during alcoholic fermentation
Source: PLoS One. 2019 Jul 26;14(7):e0220515. doi: 10.1371/journal.pone.0220515 (PMC6660096; doi:10.1371/journal.pone.0220515)
Supplement: S4 Table — (PDF) [file pone.0220515.s011.pdf]

## S4 Table. Nitrogen consumption (mgN/L) for *GTR1* reciprocal hemizygous strains.

| Nitrogen Source | WA *gtr1*Δ x WE | | WA x WE *gtr1*Δ | | ANOVA p-value | WA *gtr1*Δ x NA | | WA x NA *gtr1*Δ | | ANOVA p-value | WA *gtr1*Δ x SA | | WA x SA *gtr1*Δ | | ANOVA p-value |
|---|---|---|---|---|---|---|---|---|---|---|---|---|---|---|---|
| | Mean | SD | Mean | SD | | Mean | SD | Mean | SD | | Mean | SD | Mean | SD | |
| Aspartic | 3.420 | 0.031 | 3.638 | 0.064 | **0.0060** | 3.164 | 0.017 | 3.114 | 0.037 | 0.100 | 3.073 | 0.015 | 3.418 | 0.047 | **0.0003** |
| Glutamic | 4.578 | 0.082 | 5.600 | 0.184 | **0.0009** | 2.310 | 0.232 | 2.897 | 0.175 | **0.025** | 3.252 | 0.215 | 4.357 | 0.144 | **0.0018** |
| Serine | 6.008 | 0.025 | 6.609 | 0.122 | **0.0011** | 6.398 | 0.054 | 6.315 | 0.076 | 0.199 | 4.997 | 0.008 | 5.499 | 0.076 | **0.0003** |
| Histidine | 3.672 | 0.066 | 3.113 | 0.058 | **0.0004** | 3.244 | 0.112 | 3.121 | 0.103 | 0.235 | 2.836 | 0.036 | 2.942 | 0.077 | 0.0976 |
| Glutamine | 31.316 | 0.292 | 32.470 | 0.458 | **0.0212** | 32.500 | 0.588 | 31.556 | 0.383 | 0.080 | 29.166 | 0.175 | 30.945 | 0.293 | **0.0008** |
| Glycine | -0.251 | 0.047 | -0.264 | 0.042 | 0.7466 | 0.041 | 0.067 | 0.112 | 0.088 | 0.322 | -0.151 | 0.030 | -0.234 | 0.036 | **0.0372** |
| Arginine | 6.954 | 0.037 | 6.425 | 0.223 | **0.0155** | 7.028 | 0.393 | 6.892 | 0.390 | 0.693 | 6.332 | 0.076 | 6.593 | 0.152 | 0.0564 |
| Threonine | 6.881 | 0.052 | 7.491 | 0.097 | **0.0007** | 7.533 | 0.070 | 7.425 | 0.030 | 0.069 | 6.292 | 0.010 | 6.840 | 0.054 | **< 0.0001** |
| Alanine | 3.513 | 0.210 | 5.177 | 0.246 | **0.0009** | 3.781 | 0.711 | 4.494 | 0.493 | 0.226 | 2.963 | 0.165 | 3.658 | 0.248 | **0.0155** |
| Tyrosine | 0.998 | 0.019 | 1.156 | 0.006 | **0.0002** | 0.919 | 0.044 | 0.918 | 0.017 | 0.982 | 0.954 | 0.018 | 1.082 | 0.017 | **0.0008** |
| Valine | 5.127 | 0.015 | 5.330 | 0.021 | **0.0002** | 4.901 | 0.068 | 4.943 | 0.069 | 0.497 | 5.033 | 0.018 | 5.266 | 0.008 | **< 0.0001** |
| Methionine | ND | ND | ND | ND | | ND | ND | ND | ND | | ND | ND | ND | ND | |
| Cysteine | 0.508 | 0.037 | 0.568 | 0.061 | 0.2199 | 0.672 | 0.112 | 0.527 | 0.013 | 0.090 | 0.595 | 0.060 | 0.493 | 0.028 | 0.0552 |
| Tryptophane | 7.597 | 0.290 | 7.927 | 0.045 | 0.1226 | 7.696 | 0.472 | 7.930 | 0.306 | 0.511 | 10.009 | 0.440 | 9.757 | 0.103 | 0.3891 |
| Isoleucine | 3.951 | 0.013 | 3.936 | 0.015 | 0.2496 | 3.906 | 0.008 | 3.915 | 0.017 | 0.477 | 3.936 | 0.012 | 3.955 | 0.007 | 0.0775 |
| Leucine | 5.232 | 0.010 | 5.206 | 0.018 | 0.0896 | 5.226 | 0.011 | 5.211 | 0.014 | 0.213 | 5.210 | 0.002 | 5.217 | 0.003 | **0.0380** |
| Phenilalanine | 5.291 | 0.024 | 5.282 | 0.024 | 0.6690 | 5.096 | 0.035 | 5.104 | 0.043 | 0.808 | 5.212 | 0.011 | 5.286 | 0.003 | **0.0003** |
| Lysine | 1.970 | 0.028 | 1.930 | 0.035 | 0.1991 | 1.912 | 0.008 | 1.944 | 0.014 | **0.023** | 1.910 | 0.012 | 1.945 | 0.027 | 0.1112 |
| Ammonium | 73.044 | 2.073 | 41.632 | 1.649 | **< 0.0001** | 53.417 | 2.511 | 48.992 | 2.268 | 0.086 | 54.456 | 2.260 | 49.814 | 3.388 | 0.1196 |
| Total aminoacids | 99.354 | 0.113 | 104.182 | 0.552 | **0.0001** | 98.910 | 1.610 | 99.007 | 1.600 | 0.945 | 94.207 | 0.395 | 99.606 | 0.848 | **0.0006** |

ND: Not determined
