# Supplementary material for: Genetic variants of TORC1 signaling pathway affect nitrogen consumption in Saccharomyces cerevisiae during alcoholic fermentation
Source: PLoS One. 2019 Jul 26;14(7):e0220515. doi: 10.1371/journal.pone.0220515 (PMC6660096; doi:10.1371/journal.pone.0220515)
Supplement: S5 Table — (PDF) [file pone.0220515.s012.pdf]

S5 Table. Nitrogen consumption (mgN/L) for *SCH9* reciprocal hemizygous strains.

Nitrogen Source	WA *sch9Δ* x WE		WA x WE *sch9Δ*		ANOVA p-value	WA *sch9Δ* x NA		WA x NA *sch9Δ*		ANOVA p-value	WA *sch9Δ* x SA		WA x SA *sch9Δ*		ANOVA p-value
	**Mean**	**SD**	**Mean**	**SD**		**Mean**	**SD**	**Mean**	**SD**		**Mean**	**SD**	**Mean**	**SD**	
Aspartic	3.382	0.056	3.309	0.056	0.1884	3.199	0.023	2.955	0.032	**0.0004**	3.009	0.029	2.998	0.053	0.7695
Glutamic	4.391	0.240	4.354	0.063	0.8109	3.073	0.083	2.842	0.023	**0.0098**	2.922	0.124	3.189	0.077	**0.0338**
Serine	6.449	0.121	6.168	0.187	0.0944	6.587	0.087	6.104	0.093	**0.0028**	5.491	0.063	5.397	0.157	0.3902
Histidine	3.029	0.047	2.867	0.106	0.0730	2.877	0.034	2.637	0.015	**0.0004**	2.482	0.096	2.548	0.096	0.4480
Glutamine	30.656	0.485	29.791	0.640	0.1353	30.027	0.267	28.388	0.486	**0.0069**	27.782	0.306	27.750	0.462	0.9251
Glycine	-0.222	0.099	-0.253	0.063	0.6685	-0.010	0.049	0.099	0.067	0.0862	-0.005	0.044	0.083	0.026	**0.0397**
Arginine	5.676	0.958	5.407	0.244	0.6623	5.483	0.159	4.934	0.147	**0.0118**	4.723	0.139	5.438	0.266	**0.0145**
Threonine	7.074	0.067	6.894	0.154	0.1376	7.250	0.100	6.899	0.121	**0.0180**	6.498	0.126	6.433	0.093	0.5142
Alanine	3.783	0.618	3.450	0.318	0.4537	4.957	0.069	4.355	0.242	**0.0144**	3.417	0.113	3.745	0.168	**0.0484**
Tyrosine	0.947	0.027	0.957	0.017	0.5913	0.904	0.013	0.818	0.020	**0.0033**	0.865	0.005	0.909	0.019	**0.0185**
Valine	4.693	0.056	4.714	0.016	0.5694	4.562	0.007	4.353	0.043	**0.0012**	4.514	0.033	4.551	0.025	0.1934
Methionine	ND	ND	ND	ND		ND	ND	ND	ND		ND	ND	ND	ND	
Cysteine	0.282	0.202	0.309	0.018	0.8271	0.207	0.034	0.169	0.086	0.5100	0.147	0.042	0.342	0.050	**0.0067**
Tryptophane	7.449	0.245	7.367	0.350	0.7565	7.429	0.260	7.777	0.429	0.2957	10.164	0.084	10.576	0.347	0.1159
Isoleucine	3.673	0.019	3.681	0.008	0.5000	3.664	0.002	3.617	0.012	**0.0027**	3.653	0.007	3.642	0.004	0.1003
Leucine	4.927	0.040	4.950	0.032	0.4680	4.999	0.007	4.959	0.019	**0.0289**	4.977	0.015	4.925	0.009	**0.0078**
Phenilalanine	3.101	0.034	3.121	0.018	0.3999	3.007	0.012	2.910	0.026	**0.0042**	3.033	0.019	3.036	0.011	0.8214
Lysine	1.711	0.014	1.741	0.009	**0.0336**	1.738	0.018	1.737	0.037	0.9574	1.727	0.016	1.711	0.008	0.2116
Ammonium	55.752	1.190	51.668	2.196	**0.0472**	44.191	0.563	43.100	1.728	0.3572	43.466	1.797	48.242	4.359	0.1542
Total aminoacids	93.484	2.991	91.314	0.716	0.2888	92.441	0.897	88.038	0.710	**0.0026**	87.883	0.617	89.760	1.496	0.1148

ND: Not determined
