# Supplementary material for: Genetic variants of TORC1 signaling pathway affect nitrogen consumption in Saccharomyces cerevisiae during alcoholic fermentation
Source: PLoS One. 2019 Jul 26;14(7):e0220515. doi: 10.1371/journal.pone.0220515 (PMC6660096; doi:10.1371/journal.pone.0220515)
Supplement: S6 Table — (PDF) [file pone.0220515.s013.pdf]

S6 Table. Nitrogen consumption (mgN/L) for *SAP185* reciprocal hemizygous strains.

Nitrogen Source	WA *sap185Δ* x WE		WA x WE *sap185Δ*		ANOVA p-value	WA *sap185Δ* x NA		WA x NA *sap185Δ*		ANOVA p-value	WA *sap185Δ* x SA		WA x SA *sap185Δ*		ANOVA p-value
	Mean	SD	Mean	SD		Mean	SD	Mean	SD		Mean	SD	Mean	SD	
Aspartic	3.488	0.055	3.343	0.092	0.0806	3.326	0.015	3.022	0.106	**0.0080**	3.513	0.050	3.745	0.029	**0.0023**
Glutamic	4.858	0.121	4.825	0.090	0.7269	3.823	0.040	2.854	0.156	**0.0005**	4.183	0.266	4.089	0.127	0.6098
Serine	6.429	0.095	6.016	0.213	**0.0374**	6.664	0.033	6.478	0.248	0.2672	6.352	0.094	6.330	0.043	0.7354
Histidine	3.175	0.121	3.094	0.063	0.3637	3.114	0.031	2.773	0.192	**0.0386**	3.136	0.126	3.109	0.032	0.7365
Glutamine	32.242	0.407	31.026	0.986	0.1196	33.040	0.316	31.208	0.999	**0.0388**	33.054	0.616	33.235	0.287	0.6687
Glycine	-0.068	0.063	-0.144	0.065	0.2206	0.140	0.040	0.276	0.029	**0.0090**	-0.009	0.023	-0.350	0.022	**< 0.0001**
Arginine	6.642	0.200	6.487	0.214	0.4126	6.939	0.281	6.495	0.213	0.0945	7.347	0.332	7.331	0.073	0.9364
Threonine	7.405	0.082	6.945	0.184	**0.0167**	7.689	0.028	7.236	0.207	**0.0198**	7.481	0.082	7.301	0.015	**0.0199**
Alanine	5.053	0.180	4.083	0.084	**0.0011**	5.459	0.045	3.862	0.094	**< 0.0001**	5.301	0.218	5.269	0.098	0.8278
Tyrosine	1.018	0.016	0.964	0.013	**0.0102**	0.962	0.025	0.677	0.028	**0.0002**	1.089	0.020	1.167	0.005	**0.0028**
Valine	5.298	0.031	5.193	0.010	**0.0052**	5.193	0.028	4.509	0.108	**0.0004**	5.321	0.003	5.302	0.006	**0.0062**
Methionine	ND	ND	ND	ND		ND	ND	ND	ND		ND	ND	ND	ND	
Cysteine	0.497	0.065	0.544	0.046	0.3683	0.646	0.056	0.469	0.019	**0.0066**	0.725	0.164	0.587	0.045	0.2337
Tryptophane	7.151	0.157	7.322	0.160	0.2559	6.663	0.027	7.904	0.124	**< 0.0001**	8.969	0.360	11.236	0.165	**0.0006**
Isoleucine	3.957	0.009	3.942	0.011	0.1391	3.943	0.012	3.869	0.021	**0.0057**	3.946	0.017	3.958	0.001	0.2891
Leucine	5.197	0.029	5.222	0.005	0.2172	5.234	0.003	5.257	0.000	**0.0002**	5.215	0.006	5.235	0.002	**0.0063**
Phenilalanine	5.244	0.017	5.277	0.011	**0.0454**	5.142	0.015	4.859	0.065	**0.0018**	5.241	0.021	5.305	0.005	**0.0072**
Lysine	1.947	0.020	1.943	0.020	0.8017	1.946	0.023	1.900	0.029	0.1005	1.912	0.058	1.893	0.020	0.6078
Ammonium	46.722	3.123	49.986	1.238	0.1677	40.379	1.325	54.968	5.210	**0.0093**	51.457	4.349	54.895	1.077	0.2545
Total aminoacids	102.093	0.956	98.666	1.519	**0.0298**	102.501	0.832	96.215	2.054	**0.0080**	105.354	1.452	107.328	0.470	0.0887

ND: Not determined
