# Supplementary material for: Genetic variants of TORC1 signaling pathway affect nitrogen consumption in Saccharomyces cerevisiae during alcoholic fermentation
Source: PLoS One. 2019 Jul 26;14(7):e0220515. doi: 10.1371/journal.pone.0220515 (PMC6660096; doi:10.1371/journal.pone.0220515)
Supplement: S7 Table — (PDF) [file pone.0220515.s014.pdf]

S7 Table. Nitrogen consumption (mgN/L) for *SIT4* reciprocal hemizygous strains.

Nitrogen Source	WA *sit4*Δ x WE		WA x WE *sit4*Δ		ANOVA p-value	WA *sit4*Δ x NA		WA x NA *sit4*Δ		ANOVA p-value	WA *sit4*Δ x SA		WA x SA *sit4*Δ		ANOVA p-value
	**Mean**	**SD**	**Mean**	**SD**		**Mean**	**SD**	**Mean**	**SD**		**Mean**	**SD**	**Mean**	**SD**	
Aspartic	3.462	0.089	3.449	0.069	0.8550	3.270	0.008	3.115	0.078	**0.0268**	3.133	0.064	3.020	0.015	**0.0406**
Glutamic	3.851	0.389	4.059	0.119	0.4244	2.797	0.014	3.065	0.208	0.0903	2.469	0.090	1.938	0.230	**0.0205**
Serine	7.292	0.218	6.969	0.240	0.1600	7.076	0.051	6.638	0.185	**0.0168**	6.625	0.157	6.204	0.059	**0.0122**
Histidine	3.108	0.041	3.257	0.110	0.0941	3.074	0.030	2.881	0.133	0.0698	2.853	0.098	2.632	0.136	0.0841
Glutamine	32.379	0.839	32.062	0.923	0.6829	31.308	0.090	30.763	0.682	0.2419	29.471	0.534	29.195	0.422	0.5219
Glycine	0.163	0.142	0.030	0.076	0.2277	0.260	0.023	0.487	0.071	**0.0062**	0.349	0.057	0.335	0.080	0.8126
Arginine	6.744	1.328	6.267	0.958	0.6404	5.939	0.121	6.603	0.759	0.2091	6.060	0.129	6.308	0.389	0.3534
Threonine	7.518	0.109	7.331	0.146	0.1494	7.508	0.034	7.334	0.124	0.0787	6.982	0.108	6.705	0.073	**0.0213**
Alanine	5.350	0.723	3.869	0.408	**0.0366**	5.668	0.178	5.293	0.396	0.2091	4.891	0.254	3.252	0.227	**0.0011**
Tyrosine	0.845	0.017	0.896	0.009	**0.0100**	0.823	0.015	0.793	0.023	0.1355	0.691	0.013	0.623	0.006	**0.0010**
Valine	4.243	0.020	4.353	0.027	**0.0048**	4.458	0.032	4.566	0.033	**0.0154**	3.681	0.034	3.241	0.015	**< 0.0001**
Methionine	ND	ND	ND	ND		ND	ND	ND	ND		ND	ND	ND	ND	
Cysteine	0.800	0.338	0.717	0.206	0.7358	0.611	0.071	0.768	0.174	0.2221	0.665	0.128	0.732	0.104	0.5200
Tryptophane	7.587	0.362	8.307	0.117	**0.0308**	7.178	0.294	6.601	0.192	**0.0463**	10.549	0.366	11.375	0.714	0.1491
Isoleucine	3.668	0.004	3.682	0.010	0.0825	3.703	0.012	3.712	0.009	0.3742	3.500	0.025	3.339	0.005	**0.0004**
Leucine	4.898	0.008	4.856	0.008	**0.0026**	4.885	0.025	4.835	0.028	0.0842	4.828	0.032	4.778	0.019	0.0811
Phenilalanine	3.056	0.006	3.071	0.011	0.1183	2.948	0.025	2.825	0.027	**0.0045**	2.888	0.024	2.744	0.010	**0.0007**
Lysine	1.741	0.016	1.692	0.009	**0.0096**	1.747	0.042	1.760	0.014	0.6443	1.666	0.002	1.670	0.036	0.8438
Ammonium	68.427	4.343	75.383	5.033	0.1441	60.560	0.970	54.581	2.973	**0.0296**	71.636	2.796	74.315	2.082	0.2540
Total aminoacids	99.039	4.450	97.202	3.154	0.5910	95.587	0.632	94.353	2.927	0.5149	93.636	1.484	90.426	0.549	**0.0246**

ND: Not determined
