# Supplementary material for: Genetic variants of TORC1 signaling pathway affect nitrogen consumption in Saccharomyces cerevisiae during alcoholic fermentation
Source: PLoS One. 2019 Jul 26;14(7):e0220515. doi: 10.1371/journal.pone.0220515 (PMC6660096; doi:10.1371/journal.pone.0220515)
Supplement: S8 Table — (PDF) [file pone.0220515.s015.pdf]

S8 Table. Nitrogen consumption (mgN/L) for *TOR2* reciprocal hemizygous strains.

Nitrogen Source	WA *tor2*Δ x WE		WA x WE *tor2*Δ		ANOVA p-value	WA *tor2*Δ x NA		WA x NA *tor2*Δ		ANOVA p-value	WA *tor2*Δ x SA		WA x SA *tor2*Δ		ANOVA p-value
	Mean	SD	Mean	SD		Mean	SD	Mean	SD		Mean	SD	Mean	SD	
Aspartic	3.265	0.020	3.257	0.030	0.7213	3.114	0.064	3.031	0.047	0.1463	3.060	0.034	2.827	0.065	**0.0054**
Glutamic	3.937	0.195	3.419	0.350	0.0886	2.829	0.133	2.968	0.154	0.3010	1.821	0.259	1.583	0.169	0.2528
Serine	5.754	0.137	5.760	0.127	0.9629	6.036	0.135	5.877	0.133	0.2206	6.398	0.093	6.086	0.129	**0.0271**
Histidine	2.914	0.084	2.896	0.060	0.7743	2.723	0.032	2.508	0.085	**0.0150**	2.712	0.046	2.391	0.050	**0.0012**
Glutamine	28.900	0.557	28.637	0.282	0.5072	29.704	0.575	28.768	0.434	0.0876	29.553	0.135	28.328	0.551	**0.0201**
Glycine	0.249	0.055	0.163	0.083	0.2113	0.193	0.020	0.308	0.044	**0.0148**	0.277	0.074	0.423	0.011	**0.0280**
Arginine	7.345	0.266	6.440	0.623	0.0815	6.032	0.302	6.226	0.471	0.5808	6.454	0.223	6.089	0.371	0.2183
Threonine	6.046	0.111	6.100	0.067	0.5125	6.801	0.116	6.641	0.088	0.1299	6.951	0.055	6.724	0.107	**0.0309**
Alanine	3.213	0.118	2.543	0.600	0.1307	3.186	0.185	3.623	0.321	0.1107	3.729	0.369	3.408	0.252	0.2804
Tyrosine	0.848	0.012	0.796	0.017	**0.0121**	0.785	0.003	0.803	0.013	0.0728	0.712	0.033	0.622	0.011	**0.0103**
Valine	4.309	0.092	4.118	0.041	**0.0301**	4.396	0.011	4.472	0.027	**0.0108**	3.645	0.103	3.242	0.064	**0.0045**
Methionine	ND	ND	ND	ND		ND	ND	ND	ND		ND	ND	ND	ND	
Cysteine	0.921	0.064	0.625	0.155	**0.0377**	0.544	0.092	0.747	0.146	0.1106	0.731	0.074	0.698	0.078	0.6242
Tryptophane	10.951	0.108	11.351	0.028	**0.0034**	9.928	0.396	10.293	0.251	0.2490	11.676	0.423	12.257	0.216	0.1014
Isoleucine	3.668	0.016	3.634	0.011	**0.0393**	3.668	0.008	3.692	0.012	**0.0420**	3.503	0.027	3.302	0.029	**0.0009**
Leucine	4.863	0.001	4.845	0.007	**0.0092**	4.856	0.003	4.858	0.015	0.8019	4.866	0.015	4.708	0.038	**0.0025**
Phenilalanine	3.086	0.008	3.058	0.007	**0.0092**	2.920	0.008	2.942	0.015	0.0985	2.931	0.009	2.727	0.031	**0.0004**
Lysine	1.582	0.027	1.570	0.015	0.5311	1.694	0.004	1.731	0.007	**0.0016**	1.652	0.032	1.647	0.052	0.8935
Ammonium	87.530	4.340	84.706	7.618	0.6067	73.515	2.641	67.085	2.327	**0.0340**	78.567	1.622	72.995	0.959	**0.0069**
Total aminoacids	90.750	2.250	96.639	2.996	0.0529	91.744	1.162	91.825	2.134	0.9569	93.007	1.795	89.395	1.654	0.0624

ND: Not determined
