# Supplementary material for: Genetic variants of TORC1 signaling pathway affect nitrogen consumption in Saccharomyces cerevisiae during alcoholic fermentation
Source: PLoS One. 2019 Jul 26;14(7):e0220515. doi: 10.1371/journal.pone.0220515 (PMC6660096; doi:10.1371/journal.pone.0220515)
Supplement: S9 Table — (PDF) [file pone.0220515.s016.pdf]

S9 Table. Nitrogen consumption (mgN/L) for *NPR1* reciprocal hemizygous strains.

Nitrogen Source	WA *npr1Δ* x WE		WA x WE *npr1Δ*		ANOVA p-value	WA *npr1Δ* x NA		WA x NA *npr1Δ*		ANOVA p-value	WA *npr1Δ* x SA		WA x SA *npr1Δ*		ANOVA p-value
	**Mean**	**SD**	**Mean**	**SD**		**Mean**	**SD**	**Mean**	**SD**		**Mean**	**SD**	**Mean**	**SD**	
Aspartic	3.331	0.050	3.309	0.008	0.4970	3.621	0.005	3.387	0.063	**0.0030**	3.269	0.010	3.190	0.118	0.3106
Glutamic	5.872	0.101	5.736	0.044	0.0984	6.136	0.278	5.130	0.126	**0.0046**	5.175	0.194	5.402	0.544	0.5331
Serine	6.224	0.138	6.140	0.080	0.4099	7.213	0.021	6.782	0.152	**0.0083**	5.111	0.023	5.141	0.395	0.9029
Histidine	2.906	0.062	2.821	0.093	0.2573	3.128	0.028	2.975	0.081	**0.0363**	2.937	0.133	2.873	0.101	0.5448
Glutamine	27.997	0.620	27.840	0.329	0.7182	31.144	0.093	30.156	0.455	**0.0211**	25.843	0.147	26.362	1.327	0.5375
Glycine	-0.113	0.032	-0.182	0.069	0.1915	0.039	0.086	-0.192	0.089	**0.0315**	-0.200	0.045	0.071	0.229	0.1142
Arginine	3.992	0.188	3.876	0.150	0.4490	6.137	0.820	4.598	0.762	0.0760	4.398	0.307	5.982	1.744	0.1962
Threonine	6.766	0.037	6.679	0.047	0.0659	7.266	0.022	7.092	0.094	**0.0358**	5.487	0.029	5.509	0.292	0.9014
Alanine	3.489	0.063	3.235	0.094	**0.0178**	4.618	0.564	3.134	0.493	**0.0265**	1.865	0.413	2.558	1.159	0.3846
Tyrosine	0.936	0.030	0.938	0.002	0.9287	0.927	0.014	0.949	0.023	0.2253	0.842	0.045	0.828	0.019	0.6517
Valine	4.490	0.068	4.505	0.039	0.7566	4.021	0.048	4.147	0.092	0.1057	3.963	0.143	3.883	0.067	0.4273
Methionine	ND	ND	ND	ND		ND	ND	ND	ND		ND	ND	ND	ND	
Cysteine	-0.228	0.102	-0.248	0.052	0.7838	0.500	0.312	-0.092	0.189	**0.0482**	-0.140	0.100	0.317	0.490	0.1880
Tryptophane	7.009	0.300	6.943	0.223	0.7748	8.008	0.098	7.665	0.386	0.2099	10.343	0.317	10.701	0.341	0.2528
Isoleucine	3.200	0.021	3.214	0.007	0.3254	3.160	0.011	3.192	0.019	0.0623	3.168	0.028	3.134	0.024	0.1897
Leucine	4.998	0.062	5.030	0.024	0.4531	5.030	0.012	5.055	0.022	0.1479	5.041	0.031	4.971	0.040	0.0758
Phenilalanine	4.066	0.051	4.087	0.027	0.5536	4.083	0.011	4.123	0.022	**0.0466**	4.120	0.025	4.084	0.031	0.1962
Lysine	1.716	0.042	1.735	0.011	0.4908	1.636	0.021	1.713	0.029	**0.0205**	1.538	0.079	1.517	0.015	0.6729
Ammonium	54.842	1.819	53.687	2.326	0.5354	67.022	1.353	58.326	5.583	0.0587	77.343	5.924	78.968	5.309	0.7413
Total aminoacids	88.831	0.954	87.838	0.602	0.2020	98.848	2.262	91.994	1.913	**0.0160**	84.939	1.125	88.704	6.374	0.3707

ND: Not determined
