# Supplementary material for: Genetic variants of TORC1 signaling pathway affect nitrogen consumption in Saccharomyces cerevisiae during alcoholic fermentation
Source: PLoS One. 2019 Jul 26;14(7):e0220515. doi: 10.1371/journal.pone.0220515 (PMC6660096; doi:10.1371/journal.pone.0220515)
Supplement: S10 Table — (PDF) [file pone.0220515.s017.pdf]

**S10 Table. Nitrogen consumption (mgN/L) for *EAP1* reciprocal hemizygous strains.**

| Nitrogen Source | WA *eap1*Δ x WE | | WA x WE *eap1*Δ | | ANOVA p-value | WA *eap1*Δ x NA | | WA x NA *eap1*Δ | | ANOVA p-value | WA *eap1*Δ x SA | | WA x SA *eap1*Δ | | ANOVA p-value |
|---|---|---|---|---|---|---|---|---|---|---|---|---|---|---|---|
| | Mean | SD | Mean | SD | | Mean | SD | Mean | SD | | Mean | SD | Mean | SD | |
| Aspartic | 3.153 | 0.016 | 3.196 | 0.043 | 0.1738 | 2.871 | 0.006 | 2.892 | 0.042 | 0.4448 | 3.076 | 0.045 | 2.995 | 0.065 | 0.1505 |
| Glutamic | 3.497 | 0.136 | 3.806 | 0.261 | 0.1426 | 3.092 | 0.155 | 3.165 | 0.133 | 0.5661 | 2.649 | 0.224 | 2.435 | 0.228 | 0.3092 |
| Serine | 5.147 | 0.024 | 5.259 | 0.047 | **0.0209** | 5.112 | 0.024 | 5.156 | 0.099 | 0.4899 | 6.027 | 0.083 | 5.867 | 0.147 | 0.1751 |
| Histidine | 2.854 | 0.027 | 2.899 | 0.024 | 0.0899 | 2.520 | 0.028 | 2.579 | 0.030 | 0.0668 | 2.828 | 0.038 | 2.802 | 0.183 | 0.8212 |
| Glutamine | 27.288 | 0.044 | 27.844 | 0.143 | **0.0030** | 27.263 | 0.116 | 27.346 | 0.384 | 0.7379 | 28.883 | 0.268 | 28.203 | 0.675 | 0.1807 |
| Glycine | -0.234 | 0.017 | -0.116 | 0.057 | **0.0261** | 0.080 | 0.082 | 0.056 | 0.068 | 0.7188 | 0.075 | 0.075 | 0.067 | 0.118 | 0.9317 |
| Arginine | 5.590 | 0.206 | 6.437 | 0.463 | **0.0444** | 5.569 | 0.460 | 5.931 | 0.212 | 0.2832 | 6.461 | 0.598 | 6.289 | 0.137 | 0.6527 |
| Threonine | 5.732 | 0.063 | 5.850 | 0.024 | **0.0386** | 6.234 | 0.026 | 6.179 | 0.109 | 0.4436 | 6.554 | 0.047 | 6.384 | 0.116 | 0.0774 |
| Alanine | 2.395 | 0.176 | 3.010 | 0.406 | 0.0739 | 3.256 | 0.262 | 3.488 | 0.133 | 0.2427 | 3.772 | 0.369 | 3.633 | 0.617 | 0.7534 |
| Tyrosine | 0.885 | 0.013 | 0.922 | 0.023 | 0.0762 | 0.867 | 0.010 | 0.871 | 0.016 | 0.6874 | 0.805 | 0.051 | 0.788 | 0.040 | 0.6705 |
| Valine | 4.161 | 0.013 | 4.223 | 0.085 | 0.2786 | 4.409 | 0.012 | 4.429 | 0.025 | 0.2693 | 3.618 | 0.095 | 3.523 | 0.188 | 0.4782 |
| Methionine | ND | ND | ND | ND | | ND | ND | ND | ND | | ND | ND | ND | ND | |
| Cysteine | 0.319 | 0.034 | 0.536 | 0.145 | 0.0656 | 0.320 | 0.152 | 0.434 | 0.041 | 0.2746 | 0.479 | 0.102 | 0.411 | 0.057 | 0.3700 |
| Tryptophane | 11.178 | 0.003 | 10.972 | 0.299 | 0.2986 | 10.149 | 0.093 | 10.213 | 0.785 | 0.8959 | 11.858 | 0.355 | 12.173 | 0.574 | 0.4636 |
| Isoleucine | 3.623 | 0.013 | 3.636 | 0.021 | 0.3881 | 3.628 | 0.007 | 3.640 | 0.011 | 0.1825 | 3.417 | 0.032 | 3.375 | 0.069 | 0.3904 |
| Leucine | 4.949 | 0.020 | 4.969 | 0.037 | 0.4604 | 4.949 | 0.005 | 4.959 | 0.025 | 0.5141 | 4.912 | 0.016 | 4.888 | 0.021 | 0.1781 |
| Phenilalanine | 3.143 | 0.013 | 3.171 | 0.022 | 0.1271 | 3.009 | 0.003 | 3.020 | 0.016 | 0.3060 | 2.955 | 0.019 | 2.921 | 0.050 | 0.3213 |
| Lysine | 1.587 | 0.032 | 1.625 | 0.054 | 0.3454 | 1.741 | 0.012 | 1.732 | 0.007 | 0.3354 | 1.602 | 0.016 | 1.647 | 0.026 | 0.0643 |
| Ammonium | 73.694 | 0.734 | 73.011 | 3.145 | 0.7328 | 51.283 | 1.334 | 53.647 | 0.651 | 0.0509 | 70.540 | 1.471 | 70.819 | 2.362 | 0.8706 |
| Total aminoacids | 87.754 | 0.648 | 90.727 | 1.604 | **0.0409** | 87.552 | 1.181 | 88.578 | 1.088 | 0.3306 | 92.458 | 2.146 | 90.886 | 1.305 | 0.3393 |

ND: Not determined
