# Supplementary material for: Genetic variants of TORC1 signaling pathway affect nitrogen consumption in Saccharomyces cerevisiae during alcoholic fermentation
Source: PLoS One. 2019 Jul 26;14(7):e0220515. doi: 10.1371/journal.pone.0220515 (PMC6660096; doi:10.1371/journal.pone.0220515)
Supplement: S11 Table — (PDF) [file pone.0220515.s018.pdf]

S11 Table. Nitrogen consumption (mgN/L) for hybrids strains.

Nitrogen Source	WA x WE		WA x NA		WA x SA		NA x WE		NA x SA		SA x WE	
	Mean	SD	Mean	SD	Mean	SD	Mean	SD	Mean	SD	Mean	SD
Aspartic	3.018	0.086	3.533	0.134	3.198	0.043	3.403	0.024	3.183	0.068	3.306	0.082
Glutamic	3.350	0.149	3.288	0.182	3.523	0.155	3.250	0.105	2.127	0.401	3.495	0.199
Serine	5.747	0.139	7.660	0.300	6.059	0.118	7.308	0.095	6.749	0.153	6.258	0.240
Histidine	2.685	0.106	3.006	0.107	2.606	0.072	2.743	0.021	2.556	0.115	2.740	0.095
Glutamine	30.603	0.380	33.802	1.225	30.964	0.168	31.670	0.133	30.178	0.672	29.490	0.710
Glycine	0.310	0.098	0.479	0.049	0.202	0.050	0.540	0.056	0.513	0.109	0.359	0.120
Arginine	6.685	0.563	6.538	0.554	5.832	0.366	5.963	0.091	6.206	0.640	6.348	0.797
Threonine	7.200	0.077	7.997	0.212	6.951	0.052	7.348	0.045	6.920	0.137	6.375	0.138
Alanine	3.713	0.853	6.573	0.378	4.170	0.362	4.908	0.113	4.672	0.369	3.782	0.700
Tyrosine	0.833	0.050	0.763	0.008	0.827	0.019	0.652	0.003	0.599	0.040	0.678	0.017
Valine	5.191	0.068	4.588	0.017	4.779	0.043	3.608	0.008	3.211	0.124	3.775	0.036
Methionine	ND	ND	ND	ND	ND	ND	ND	ND	ND	ND	ND	ND
Cysteine	0.721	0.142	0.432	0.079	0.514	0.088	0.619	0.073	0.609	0.229	0.664	0.269
Tryptophane	6.700	0.501	9.499	0.204	10.377	0.833	9.032	0.639	12.219	0.129	11.503	0.363
Isoleucine	3.952	0.004	3.836	0.001	3.887	0.011	3.502	0.006	3.328	0.033	3.571	0.010
Leucine	5.231	0.024	5.228	0.003	5.229	0.007	4.849	0.001	4.737	0.033	4.888	0.020
Phenilalanine	4.847	0.099	4.908	0.019	5.128	0.019	2.856	0.011	2.727	0.035	3.043	0.021
Lysine	2.014	0.028	1.941	0.014	1.922	0.046	1.736	0.030	1.693	0.042	1.616	0.009
Ammonium	28.244	2.785	58.037	3.174	50.741	3.860	72.851	1.877	72.432	4.105	79.924	4.979
Total aminoacids	95.330	3.068	106.659	3.036	98.755	1.863	96.324	1.282	94.562	1.095	94.225	3.529

ND: Not determined
